# Spring Water of an Alpine Karst Aquifer Is Dominated by a Taxonomically Stable but Discharge-Responsive Bacterial Community

**DOI:** 10.3389/fmicb.2019.00028

**Published:** 2019-02-15

**Authors:** Domenico Savio, Philipp Stadler, Georg H. Reischer, Katalin Demeter, Rita B. Linke, Alfred P. Blaschke, Robert L. Mach, Alexander K. T. Kirschner, Hermann Stadler, Andreas H. Farnleitner

**Affiliations:** ^1^Division Water Quality and Health, Department Pharmacology, Physiology and Microbiology, Karl Landsteiner University of Health Sciences, Krems an der Donau, Austria; ^2^Interuniversity Cooperation Centre for Water and Health, Vienna, Austria; ^3^Centre for Water Resource Systems, TU Wien, Vienna, Austria; ^4^Research Unit for Water Quality Management, Institute for Water Quality and Resource Management, TU Wien, Vienna, Austria; ^5^Molecular Diagnostics Group, Institute of Chemical, Environmental and Bioscience Engineering, Department of Agrobiotechnology, IFA-Tulln, TU Wien, Tulln an der Donau, Austria; ^6^Research Group for Environmental Microbiology and Molecular Diagnostics 166/5/3, Institute of Chemical, Environmental and Bioscience Engineering, TU Wien, Vienna, Austria; ^7^Institute of Hydraulic Engineering and Water Resources Management, TU Wien, Vienna, Austria; ^8^Research Division of Biochemical Technology, Institute of Chemical, Environmental and Bioscience Engineering, TU Wien, Vienna, Austria; ^9^Institute for Hygiene and Applied Immunology, Medical University of Vienna, Vienna, Austria; ^10^Department for Water Resources Management and Environmental Analytics, Institute for Water, Energy and Sustainability, Joanneum Research, Graz, Austria

**Keywords:** high-throughput 16S rRNA gene amplicon sequencing, spring water microbiome, high-discharge event, base flow, drinking water resource characterization and protection

## Abstract

Alpine karst aquifers are important groundwater resources for the provision of drinking water all around the world. Yet, due to difficult accessibility and long-standing methodological limitations, the microbiology of these systems has long been understudied. The aim of the present study was to investigate the structure and dynamics of bacterial communities in spring water of an alpine limestone karst aquifer (LKAS2) under different hydrological conditions (base vs. event flow). The study was based on high-throughput 16S rRNA gene amplicon sequencing, study design and sample selection were guided by hydrology and pollution microbiology data. Spanning more than 27 months, our analyses revealed a taxonomically highly stable bacterial community, comprising high proportions of yet uncultivated bacteria in the suspended bacterial community fraction. Only the three candidate phyla Parcubacteria (OD1), Gracilibacteria (GN02), Doudnabacteria (SM2F11) together with *Proteobacteria* and *Bacteroidetes* contributed between 70.0 and 88.4% of total reads throughout the investigation period. A core-community of 300 OTUs consistently contributed between 37.6 and 56.3% of total reads, further supporting the hypothesis of a high temporal stability in the bacterial community in the spring water. Nonetheless, a detectable response in the bacterial community structure of the spring water was discernible during a high-discharge event. Sequence reads affiliated to the class *Flavobacteriia* clearly increased from a mean proportion of 2.3% during baseflow to a maximum of 12.7% during the early phase of the studied high-discharge event, suggesting direct impacts from changing hydrological conditions on the bacterial community structure in the spring water. This was further supported by an increase in species richness (Chao1) at higher discharge. The combination of these observations allowed the identification and characterization of three different discharge classes (Q1–Q3). In conclusion, we found a taxonomically stable bacterial community prevailing in spring waters from an alpine karst aquifer over the entire study period of more than 2 years. Clear response to changing discharge conditions could be detected for particular bacterial groups, whereas the most responsive group – bacteria affiliated to the class of *Flavobacteriia* – might harbor potential as a valuable natural indicator of “system disturbances” in karst aquifers.

## Introduction

In many regions of the world, drinking water abstraction relies on raw water from alpine or mountainous karst aquifers. For example, around 50% of the entire population of Austria (Europe) is supplied from alpine karst aquifers – including also its capital Vienna with ∼1.8 million citizens (Kralik, 2001; Stadler et al., 2008). Consequently, this holds major challenges regarding the management of these vulnerable water resources and their sustainable use. This applies particularly to regions where treatment and disinfection efforts of raw water are intentionally kept at a minimum to meet the consumer’s desire for high-quality, safely consumable drinking water without noticeable change in taste as a result of high chlorine concentrations for disinfection (Griebler and Avramov, 2015; Savio et al., 2018).

Concerning the microbiology of karst aquifers in general, and *alpine* karst aquifers in particular, the existing knowledge today is still highly restricted (Griebler and Lueders, 2009; Savio et al., 2018). This is particularly true for the *phreatic* (i.e., water-saturated) zone of *alpine* karst aquifers, while considerably more studies are available on the microbiology of the *vadose*, unsaturated zone of karstic systems, including stagnant rock-pools, caves and epigenic cave streams (Shabarova and Pernthaler, 2010; Kostanjšek et al., 2013; Shabarova et al., 2013, 2014; Brannen-Donnelly and Engel, 2015; Wu et al., 2015; Pleše et al., 2016; Tomczyk-Żak and Zielenkiewicz, 2016). For a lowland limestone aquifer in central Germany, very recent studies demonstrated a high potential for chemolithoautotrophic metabolic processes, including denitrification linked to the oxidation of reduced sulfur compounds as well as the capacity for anaerobic ammonium oxidation (Opitz et al., 2014; Herrmann et al., 2015, 2017; Kumar et al., 2017, 2018; Starke et al., 2017). A high potential for chemolithoautotrophic processes has also been suggested based on a strong contribution of dissolved inorganic carbon to the build-up of groundwater microbial biomass (Nowak et al., 2017; Schwab et al., 2017). Hence, these studies further support the perception of a generally high importance of chemolithoautotrophic processes in groundwater aquifers as already previously proposed for a shallow alluvial groundwater aquifer featuring the potential for anaerobic ammonium oxidation (Jewell et al., 2016).

Most available studies investigating the microbiology of the *phreatic* zone of *alpine* karst aquifers focused only on fecal pollution based on traditional cultivation-based methods (Stadler et al., 2008; Farnleitner et al., 2010; Sinreich et al., 2014). Moreover, in recent years, also molecular-biological methods such as quantitative PCR have increasingly been applied for the detection of overall fecal pollution on the one hand, and for the purpose of identifying the probable origin of fecal pollution based on specific Microbial Source Tracking (MST)-assays on the other hand (Reischer et al., 2006, 2007, 2008, 2011). Yet, these methods do not allow for the comprehensive study of the structure and function of the bulk natural microbial community. Consequently, only very few studies focused on members of the natural bacterial community in these pristine and hardly accessible *alpine* ecosystems (Griebler and Lueders, 2009). Although considering only small and non-representative subsets of the bulk communities due to cultivation biases (Staley and Konopka, 1985), very early cultivation-based studies already suggested an abundant microbial community within these oligotrophic, and in terms of temperature (often consistently low temperatures of ∼4–6°C) and hydrological dynamics (high shear stress during high-discharge events in highly weathered aquifers) hostile habitats (Pavuza, 1994; Menne, 1999). More than a decade later, first molecular-biological studies on the composition and activity of the bulk microbial communities in different *alpine* karst ecosystems were published (Farnleitner et al., 2005; Pronk et al., 2006, 2009; Wilhartitz et al., 2007, 2009). While most of these studies focused mainly on the unsaturated, epiphreatic zone such as caves or stagnant rock pools (Shabarova et al., 2013, 2014; Herrmann et al., 2015; Tomczyk-Żak and Zielenkiewicz, 2016), only one study focused deliberately on the bacterial community composition in the *phreatic*, permanently water-saturated zone of an *alpine* karst aquifer with high importance for drinking water abstraction (Farnleitner et al., 2005). These authors reported temporally stable bacterial populations suspended in the spring water of two *alpine* karst aquifer springs with different hydrogeological background and at different hydrological situations. Yet, during high-discharge conditions, these authors failed to detect an expected increase in bacterial species richness due to the infiltration of surface-associated populations from surface-habitats, as clearly indicated by increasing concentrations of fecal indicator bacteria and HPC22 counts (Reischer et al., 2008; Stadler et al., 2010). The lack of such observations in the bulk community composition was interpreted as a consequence of the relatively high detection limit of the applied DGGE-fingerprinting method, detecting only populations with relative abundances above ∼1% (Farnleitner et al., 2005). In contrast, another DGGE-based study investigating the bacterial dynamics in a karst aquifer located in the Swiss Jura Mountain area which is directly influenced by a swallow hole draining agricultural land could indeed observe detectable contributions of novel species after a precipitation event that have not been detected before under baseflow conditions (Pronk et al., 2009).

The overall aim of the present study was to investigate the temporal dynamics of the bacterial communities in spring water of an *alpine* karst aquifer in response to differing discharge conditions by high-resolution 16S rRNA gene amplicon sequencing. A set of hydrologically well-defined spring water samples from model aquifer “Limestone Karst Aquifer Spring 2” (LKAS2) was selected from an established DNA-sample bank and investigated in order to (1) determine the genetic community structure and taxonomic composition of the bulk bacterial community in the baseflow component of the aquifer, and (2) to resolve the genetic population dynamics during increased discharge and surface influence conditions (i.e., rainfall event). LKAS2 was chosen as model system because of its relatively short water residence time and resulting vulnerable character during precipitation events. In this context, a precise hydrological and microbiological characterization (i.e., discharge, chemical, physical and pollution microbiological background information) of samples as well as the comparability to previously performed studies on LKAS2 (Farnleitner et al., 2005; Wilhartitz et al., 2009) were considered of high importance.

## Materials and Methods

### Model System

The selected model system for the present study was “Limestone Karst Aquifer Spring 2” (LKAS2). LKAS2 is located in the Northern Calcareous Alps of Austria and drains an estimated catchment area of around 70 km^2^ at a mean altitude of 1380 m.a.s.l. (Farnleitner et al., 2005; Savio et al., 2018). According to D’amore et al. (1983), its aquifer represents a typical limestone karst aquifer with relatively short estimated average water residence time (Stadler and Strobl, 1997). The high degree of karstification of the aquifer feeding LKAS2 entails high dynamics in discharge with a ratio between maximum and minimum discharge (Q_max_/Q_min_) of ∼40, according to Stevanović (2015) representing a *variable* discharge regime, whereas a ratio < 10 is classified as *stable*, and > 100 as *extremely variable*. Sinreich et al. (2014) proposed a hydrogeological-microbiological classification system of karst springs linking spring dynamics with microbiological characteristics, degree of karstification and recharge type. According to this system, the LKAS2 aquifer represents a *vulnerable* aquifer, making it a suitable model system for the study of surface-related impacts on the aquifer microbiology (Farnleitner et al., 2005). In addition, a variety of background data has been made available for this particular aquifer through multiple previous studies (Dirnböck et al., 1999; Farnleitner et al., 2005; Wilhartitz et al., 2007, 2009, 2013; Reischer et al., 2008, 2011; Stadler et al., 2010; Savio et al., 2018).

### Hydrological Characterization of Study Period

The studied model aquifer LKAS2 shows an estimated average water residence time between 0.8 and 1.5 years from infiltration to discharge (Farnleitner et al., 2005; Savio et al., 2018). Response to precipitation events occurs very quickly within a few hours. The average discharge at the spring for the period between 1995 and 2006 was at 5,146 L s^−1^, whereas the maximum discharge in this period was measured on August 7, 2006 with ∼45,000 L s^−1^. In contrast, the minimum discharge ever recorded within this time span was at only ∼444 L s^−1^. The month with highest discharge based on long-term data from 1995 to 2006 was May with an average discharge of almost 12,000 L s^−1^, while lowest average discharges typically occur in January and February. During the sampling period of the present study from August 9, 2004 to December 5, 2006, the mean measured discharge was at ∼6,331 L s^−1^ and ranged from ∼872 to 45,713 L s^−1^, which was considerably higher than for the period between 1995–2000 (4,836 L s^−1^, Farnleitner et al., 2005) and 1995–2006 (5,146 L s^−1^). However, the studied period included three late summer and autumn seasons with high probability for high-discharge events, and “misses” one winter/spring season with typically lower probability for such events.

### Study Design and Sample Selection Based on Hydrological, Physical and Chemical Parameters

**FIGURE 1:**
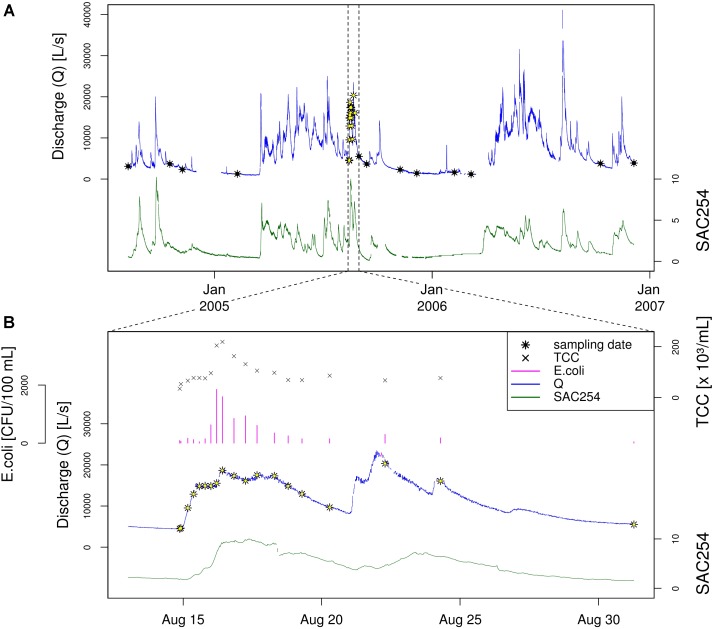
Study design. Timeline showing the sampling dates (black and black-yellow stars) along the discharge graph (blue) as well as the measured Spectral Absorption Coefficient at λ = 254 nm (SAC_254_; green) for **(A)** the entire sampling period, and **(B)** the investigated high-discharge event in August 2005. Panel **(B)** additionally depicts total cells counts (TCC) as well as concentrations for the standard fecal pollution indicator *E. coli*. Data on *E. coli* concentrations, discharge and SAC_254_ were previously published by Reischer et al. (2008).

To study the natural bacterial community in spring water of LKAS2 without the impact from allochthonous, surface-derived species, a set of samples retrieved under baseflow (BF) conditions between the years 2004 and 2006 was selected from an established DNA-sample bank. In contrast, event flow samples from a high-frequency sampling conducted during an isolated high-discharge event (EV) caused by a summer storm in August 2005 were selected to investigate precipitation-induced short-term community dynamics in the spring water with an expected import of allochthonous, surface-derived bacterial cells during high discharge. Figure 1 shows the basic study design with sampling dates and most important environmental parameters (A) for the entire sampling period and (B) for the isolated high-discharge event in particular.

To allow a reliable differentiation between BF and EV samples, selection criteria were defined regarding the prevailing hydrological conditions (discharge) as well as for turbidity and the Spectral Absorption Coefficient (SAC_254_) at the respective sampling date. For discharge, the threshold was defined at 6,000 L s^−1^ to be either categorized as baseflow (<6,000 L s^−1^) or event (>6,000 L s^−1^) sample. To eliminate unwanted effects and distortion from possible preceding high-discharge events such as recent wash-outs, the hydrograph for each sample categorized as baseflow sample was reviewed for any pronounced discharge events within 2 weeks before the respective sampling date. In hydrologically dynamic *alpine* karst aquifers such as the LKAS2 aquifer, the Spectral Absorption Coefficient at λ = 254 nm (SAC_254_) can be used as proxy for dissolved organic matter and is therefore indicative for surface-derived influence (Stadler et al., 2010), while turbidity indicates inorganic sediments that are mobilized during increasing discharge from deposits from within the system such as from conduits and caves (Stadler et al., 2010). Thresholds for these parameters were defined in order to eliminate potential impact from surface-runoff infiltration and import of surface-derived bacteria (e.g., from soils) in BF samples. Based on common spring abstraction management practice at LKAS2, the threshold for turbidity was set to a value of 0.5, while the threshold for SAC_254_ as indicator for dissolved organic surface-influence was set to 2 (Stadler et al., 2010). Impact from event-induced run-off was further indicated by microbiological indicator parameters (see following section “Physical, Chemical and Microbiological Water Quality Parameters”).

Based on this classification system, we classified 16 samples as representative for baseflow (BF), and 16 samples as representative for event (EV) conditions (*n* = 32). The selected baseflow samples (BF) were collected over a period of > 27 months, whereas baseflow samples BF01–BF08 represent samples collected before, and samples BF09-BF16 samples collected after the investigated high-discharge event (EV) sampling over ∼2 weeks (EV01–EV16). Three samples collected during the high-frequency-sampling campaign during the investigated summer storm event were characterized as BF samples (BF07, BF08, and BF09) and served as so-called “zero” or reference samples (Figure 1). Further information on the prevailing hydrological as well as physical and chemical conditions for the two sample groups, as well as for selected samples can be found in the Supplementary Material.

As previously published by Reischer et al. (2008), water samples were collected in acid- and ddH_2_0-rinsed and autoclaved Nalgene (Nalgene Europe, Hereford, UK) sampling bottles (volume 4.2 L), stored at 4°C in cooling boxes during transport and processed within 6 h after sample collection. Sample processing for molecular-biological analyses included the filtration of ∼4.2 L of spring water through 0.2 μm pore-sized polycarbonate filters (Isopore^TM^, 45 mm diameter, Millipore Corp. Bedford, MA, United States) and storage of biomass at –80°C until nucleic acid extraction. Extraction of nucleic acids from polycarbonate filters was conducted in 2007 as previously published by Reischer et al. (2008), applying a slightly modified protocol of a phenol-chloroform bead-beating-based procedure for the rapid co-extraction of DNA and RNA from natural environments by Griffiths et al. (2000), using isopropanol instead of polyethylene glycol for DNA precipitation.

### Physical, Chemical and Microbiological Water Quality Parameters

Hydrological as well as physical and chemical data were measured in 15 min intervals by on-line sensors installed directly at the spring outlet of LKAS2 as previously published (Reischer et al., 2008). The measured parameters included electrical conductivity (EC), water pressure, current, inductive discharge measurements as well as turbidity and the Spectral absorption coefficient at λ = 254 nm (SAC_254_). Median values for both turbidity and SAC_254_ were markedly higher during the event when compared to baseflow sampling dates, with a ratio of 17.1 and 9.3, respectively (Table 1). For the determination of total prokaryotic concentrations (Total Cell Counts; TCC), a slightly modified version of the Acridine orange-based direct count method after Hobbie et al. (1977) was used as previously described in Kirschner and Velimirov (1997). After fixation of up to 45 mL of spring water with buffered formaldehyde to a final concentration of 3.7% and staining with Acridine orange, TCC were determined under a Leitz Diaplan epifluorescence microscope (Leica, Wetzlar, Germany) within 14 days after storage at 4°C as described elsewhere (Wilhartitz et al., 2009, 2013). Enumeration of standard fecal indicator bacteria (SFIB) such as *Escherichia coli*, enterococci and heterotrophic plate counts at 22°C (HPC22) was previously published by Reischer et al. (2008) and conducted according to the respective ISO standard methods (ISO 9308-1:2000; ISO 7899-2:2000; ISO 6222:1999) (International Organisation for Standardisation [ISO], 1999, 2000a,b). Results from the determination of ruminant-specific BacR genetic fecal marker concentrations presented in this study were previously published by Reischer et al. (2008). The BacR-assay is a quantitative real-time PCR assay developed for the sensitive detection of the 16S rRNA gene of ruminant-specific bacteria affiliated to the phylum *Bacteroidetes* (Reischer et al., 2006). Similar to turbidity and SAC_254_, microbiological parameters such as TCC or fecal and surface indicators such as *E. coli*, genetic fecal marker BacR, and HPC22 showed pronouncedly higher concentrations during the studied high discharge event, with marked increases of TCC and *E. coli* concentrations particularly during the early phase of the investigated high-discharge event (Figure 1B). Table 1 presents descriptive statistics of selected microbiological parameters as well as ratios between event and baseflow samples as a measure of their indicator capacity for surface influence (see below).

**Table 1:** Summary table of physical, chemical and microbiological indicator parameters.

	BASEFLOW (*n* = 16)							EVENT (*n* = 16)							Reference	Ratios EV/BF	
	*n*	pos	NA	mean	median	Range	*SD*	*n*	pos	NA	mean	median	Range	*SD*		md_EV_/md_BF_	max_EV_/mean_BF_
Discharge [L s^−1^]	14	–	2	3055	3376	1173–5513	1393	16	–	0	15213	15226	9527–20336	2921		4.5	6.7
Conductivity [μS cm^−1^]	16	–	0	204	198	186–233	13	16	–	0	195	196	191–203	3		1.0	1.0
Temp [°C]	16	–	0	5.4	5.4	5.23–5.7	0.2	16	–	0	5.2	5.2	5.17–5.33	0.1		1.0	1.0
pH	16	–	0	8.0	8.1	7.5–8.2	0.2	16	–	0	7.7	7.5	7.5–8.1	0.3		0.9	1.0
SAC_254_ [abs m^−1^]	16	–	0	1.0	0.7	0.22–2.07	0.6	16	–	0	6.3	6.8	1.84–9.8	2.7	Reischer et al., 2008	9.3	9.8
Turbidity [FNU]	16	–	0	0.2	0.1	0.03–0.5	0.1	16	–	0	1.7	1.7	0.52–2.95	0.8		17.1	19.7
*E. coli* [CFU/L]	16	9	0	14.3	3.0	0–90	27	16	16	0	521.6	275.0	45–1850	543		91.7	129.8
BacR [×10^3^ ME/L]	16	14	0	1.0	0.1	0–8.4	2.3	16	16	0	149.0	48.9	2.2–820.0	221.7		350.2	840.2
TCC [×10^3^ cells/mL]	9	9	7	36.2	34.5	26–53	8	16	16	0	104.8	81.5	66–219	49		2.4	6.0

*For the calculation of ratios, all baseflow and event samples were included except for discharge and TCC, for which data were only available for 14 and 9 baseflow samples, respectively. n – sample number for which data are available; pos – positive cases/detections; NA … cases for which no data are available; md – median; SAC_254_ – Spectral Absorption Coefficient at 254 nm; ME – marker equivalents; TCC – Total cell counts/concentration.*

### Molecular-Biological Analysis of Bacterial Communities

To assess the potential presence of co-extracted inhibitory compounds, concentrations of bacterial 16S rRNA genes were quantified applying a quantitative PCR assay targeting the 16S rRNA gene of most bacteria as described previously (Savio et al., 2015). In short, quantitative PCR reactions contained 2.5 μL of 1:4.5 and 1:20.25 diluted DNA extract as the template, 0.2 μM of primers 8F (5’-AGAGTTTGATCCTGGCTCAG-3’; S-D-Bact-0008-a-S-20; Frank et al., 2007) and 338 (5’-TGCTGCCTCCCGTAGGAGT-3’; S-D-Bact-0338-a-A-19; Fierer et al., 2008) targeting the V1–V2 region of most bacterial 16S rRNA genes, and iQ^TM^ SYBR^^®^^ Green Supermix according the manufacturer’s instructions (Bio-Rad Laboratories, Hercules, CA, United States). Cycling conditions were 95°C for 3 min, 40 cycles of 95°C denaturation for 30 s, 30 s annealing at 57°C, 60 s elongation at 72°C, followed by a final elongation step at 72°C for 2 min. The ratios of measured 16S rRNA gene copy numbers between the different sample dilutions that deviated markedly from 1 after multiplication with the respective dilution factor were interpreted as an indication for PCR-inhibition. In case of observed PCR inhibition, higher dilutions (1:81 and 1:324) were prepared, 16S rRNA concentrations determined, and ratios checked again.

For the preparation of 16S rRNA gene amplicon libraries for high-resolution community analysis of 32 samples based on high-throughput amplicon sequencing, the V1–V2 region of 16S rRNA-genes in the 32 long-term stored (–80°C) DNA samples was amplified and specifically “barcoded” in a two-step barcoding-PCR procedure. In addition, six of these samples (BF03, BF06, BF12, EV06, EV12, and EV14) were technically replicated in order to investigate the reproducibility of the methodological approach (results are shown in Supplementary Figure 4). The used primers 8F (5’-AGAGTTTGATCCTGGCTCAG-3’; S-D-Bact-0008-a-S-20; Frank et al., 2007) and 338 (5’-TGCTGCCTCCCGTAGGAGT-3’; S-D-Bact-0338-a-A-19; Fierer et al., 2008) target the V1-V2 hypervariable region of bacterial 16S rRNA genes. A detailed description of the two-step barcoding-procedure can be found in the Supplementary Material and Methods. High-throughput sequencing of 16S rRNA gene amplicons was conducted on a half plate of a Genome Sequencer FLX at Selah Clinical Genomic Center (Columbia, SC, United States) using GS FLX Titanium series reagents. In total, sequencing yielded 461,460 raw sequence reads (from now on referred to as “reads”) for all 44 equimolarly pooled samples included in the run. Sequence processing and quality checking was conducted using the USEARCH pipeline (v8.1.1.1861; Edgar, 2010). The first step of sequence processing was demultiplexing of samples based on barcodes and simultaneous stripping of barcode- and primer sequences using the USEARCH pipeline (v8.1.1.1861; Edgar, 2010). 438,126 reads matched correct barcode and primer sequences. Based on information from a quality-score check using “-fastq_eestats2”-option in USEARCH, sequences were trimmed to a length of 260 bp, resulting in 347,875 reads for the dereplication-step. These sequences comprised 185,843 unique sequences from which 150,886 reads (81.2% of total unique reads) were singleton sequences. On average, each unique sequence occurred 1.87 times with a maximum occurrence of 3099. OTU clustering applying the UPARSE algorithm and integrated chimera filtering (Edgar, 2013) included only sequences with an occurrence larger than 1 and resulted in 9,242 OTUs, to which 67.3% of all preprocessed reads (292,979/435,038) could be mapped. 7,940 unique sequences (22.7% of all unique sequences with an occurrence > 1) were identified as chimera sequences. The generated OTU-table was further imported into the QIIME bioinformatics pipeline (MacQIIME version 1.9.1-2015-0604; Caporaso et al., 2010) for taxonomic classification of representative sequences using SILVA taxonomy (SILVA 123 QIIME release) (Quast et al., 2013; Yilmaz et al., 2014) and RDP classifier (Wang et al., 2007). Raw sequence data were submitted to the EMBL-EBI European Nucleotide Archive (ENA) under accession number PRJEB27895.

### Data Analysis and Statistics

Further analyses including calculation of diversity measures, statistical analyses and figure generation were conducted using the software environment “R” (R Core Team, 2014) and the R package “vegan” (Oksanen et al., 2013). To assess the diversity within samples (so-called “*alpha diversity*”), the *Chao1* richness estimator index (Chao, 1987) as well as *Pielou’s Evenness* (Pielou, 1966) were calculated to estimate the “species” richness (number of OTUs) as well as their distribution (“evenness”) within each sample. In contrast to *alpha diversity*, the concept of so-called *beta diversity* describes the diversity between different samples. For an assessment of this “between-sample diversity”, Bray–Curtis dissimilarity index was calculated (Quinn and Keough, 2002). Prior to calculation of diversity indices, OTU-tables were rarefied to a minimum number of 5,116 and 3,203 reads per sample for *alpha* and *beta diversity* analysis, respectively, whereas samples with fewer reads were excluded from the analysis. For *alpha diversity* analysis, this procedure resulted in a final number of 30 samples, while 31 samples with more than 3,203 reads were used for *beta diversity* analysis based on Bray–Curtis dissimilarity index. Non-metric multidimensional scaling (NMDS) of Bray–Curits dissimilarities was performed using the “metaMDS”-function (using default-settings allowing for square-root and Wisconsin-transformation and a setting of “trymax” to 200) implemented in the R-package “vegan” (Oksanen et al., 2013). Sample “BF05” was excluded in both, *alpha* and *beta diversity* analyses due to a too low sequence read number, not allowing for a reliable analysis. Dissimilarities between bacterial communities in different sample groups (e.g., BF vs. EV communities) were statistically tested applying PERMANOVA-analysis with 999 permutations using the “adonis”-function implemented in the R-package “vegan” (Oksanen et al., 2013). Testing of significant differences between sample groups was conducted using the non-parametric Wilcoxon rank sum test in R with default settings (“wilcox.test”; paired = FALSE, “two-sided”). “Core OTUs” were operationally defined by a minimum absolute read abundance of 100 reads and a minimum occurrence in at least 27 out of the 32 samples in total. First-occurrence analysis based on a self-written R-script (see Supplementary Material) determines the number of novel OTUs occurring for the first time in every single sample and is therefore highly dependent on the initial sample-order. Here, samples were sorted in chronological order according to the date of sampling.

### Estimated Cell Concentrations of Different Taxonomic Groups

For the analysis of absolute dynamics in community composition rather than only changes in relative abundance, an estimate for absolute cell concentrations was calculated for different bacterial taxonomic groups by multiplication of TCC with relative abundance of the taxon of interest (TCC × %_taxon_; e.g., class *Flavobacteriia*) as retrieved by 16S rRNA gene amplicon sequencing. These concentrations are referred to as “estimated taxon cell concentrations.” The calculation of estimated taxon cell concentrations was possible for all 16 event samples, whereas only 9 baseflow samples could be included due to missing TCC measurements. In this regard, we are fully aware of potential biases related to differential primer-specificity/coverage and therefore consider the obtained concentrations as coarse estimates. Yet, assuming a rather stable community without the import of large numbers of bacterial cells that are not covered by the used primers (e.g., during a high-discharge event), a between-sample comparison should indeed be valid.

### Indicator Capacity of Different Taxonomic Groups

For an assessment of the capacity of different bacterial groups to indicate recent surface-influence, the ratio between the maximum estimated cell concentrations during the event sampling (max_EV_) and the mean estimated cell concentration during baseflow (mean_BF_) was calculated and compared with values for well-known surface indicators such as *E. coli*, enterococci, HPC37 as well as for the genetic fecal marker BacR. When including all event and those nine baseflow samples for which TCC were available, the max_EV_/mean_BF_ ratios for these indicators ranged between 100 and 506 (Supplementary Table 3).

## Results

### Alpha and Beta Diversity in Response to Different Discharge Conditions

**FIGURE 2:**
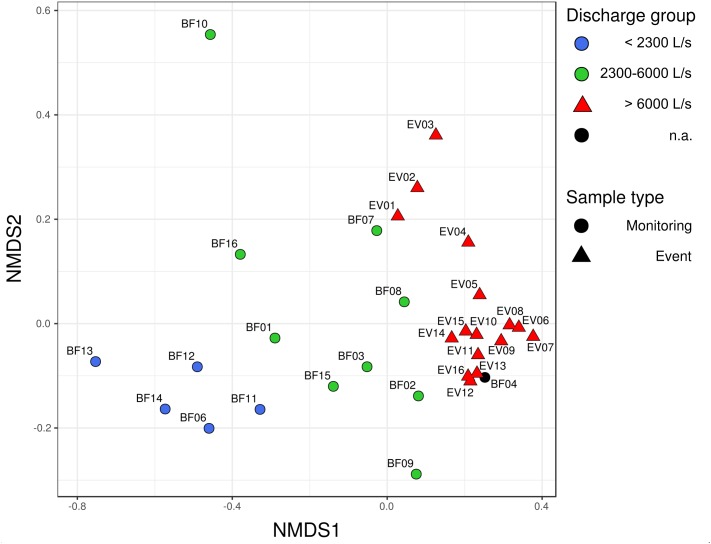
Non-metric multidimensional scaling (NMDS)-plot of community dissimilarities based on the Bray–Curtis dissimilarity index. Stress value of the NMDS was 0.10. Circles depict bacterial communities occurring under baseflow conditions (BF), triangles represent bacterial communities observed under high-discharge event conditions (EV).

**FIGURE 3:**
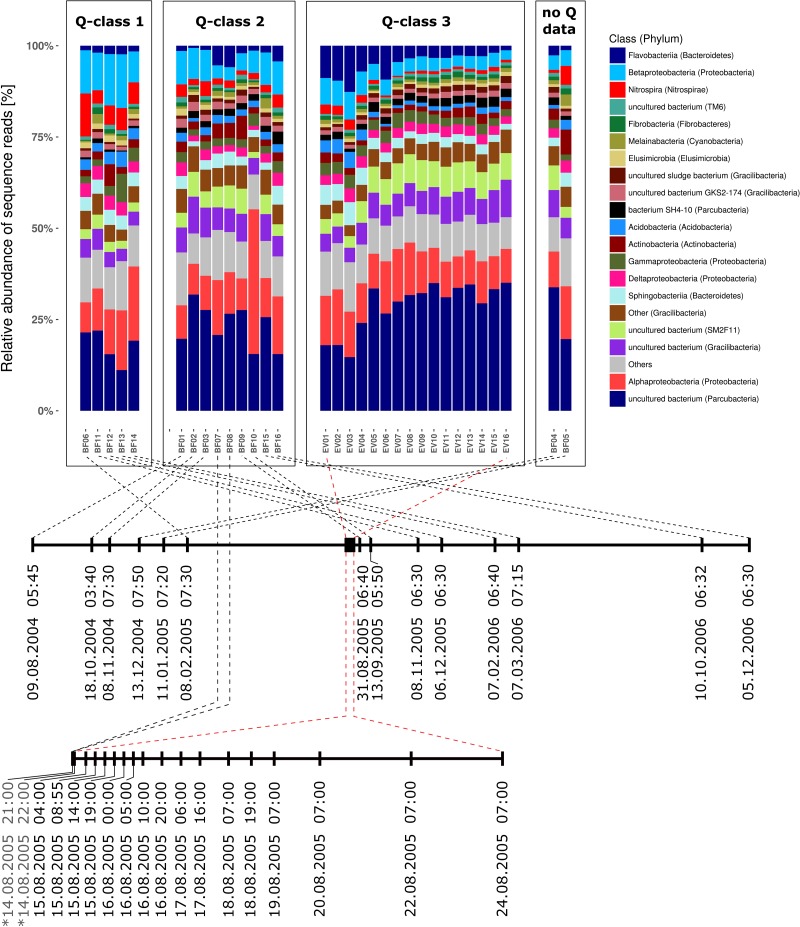
Taxonomic community composition in all investigated samples separated by discharge class (Q1–Q3) and sorted by time of sampling from left to right. Barstacks depict the relative abundance of the 20 most abundant bacterial classes according to SILVA taxonomy (v123; Yilmaz et al., 2014) as well as all additional classes summarized under “*Others*.” Brackets contain the respective phylum the bacterial class is affiliated to. Dates signed with a “^∗^” in the lower timeline (EV-window) indicate baseflow samples (BF07 and BF08).

Community dissimilarity analysis based on the Bray–Curtis dissimilarity-index measure and NMDS scaling revealed a clear spatial separation of bacterial communities between BF and EV samples (Figure 2; PERMANOVA *R*^2^ = 0.14, *p*-value 0.001). Moreover, a separation could also be observed among the BF sample group itself, whereas the two identified BF sub-clusters did not appear to follow temporal trends, i.e., whether sampling was conducted before (BF01–BF08) or after the investigated high-discharge event (BF09–BF16, see Figure 2, 3). Instead, their separation rather followed the prevailing discharge conditions on the respective sampling date. Based on this observation, three discharge classes associated with different bacterial communities could be defined: discharge-class 1 (Q1) at < 2,300 L s^−1^, discharge class 2 (Q2) ranging from 2,300 to 6,000 L s^−1^, and discharge class 3 (Q3) at > 6,000 L s^−1^ (Figure 2). Only four samples, namely BF04, BF07, BF08 and BF10, did not classify according to this operationally defined community-classification based on NMDS-visualization of community dissimilarities, whereas only the latter was considered a clear outlier (see discussion in Supplementary Material). Within the event-sample communities, samples EV01–EV03 – representative for the early phase of the event – separate slightly from the remainder EV samples in the multidimensional space (Figure 2).

For all three determined *alpha diversity* indices – the number of observed species (S.obs), *Chao1* richness estimator (Chao1) and *Pielou’s Evenness* (J) – values in the EV samples were significantly increased when compared to BF samples as tested by Wilcoxon rank sum test (S.obs, *p* = 0.002; Chao1, *p* = 0.01; J, *p* < 0.001). The classification of three different community groups corresponding to the operationally defined discharge groups Q1–Q3 was also well-reflected in the results from *alpha diversity* estimation. Here, a clear increase in the estimated number of OTUs based on Chao1 richness estimation and Pielou’s Evenness could be observed with increasing discharge (Supplementary Figure 1). All three diversity metrics were lowest under low discharge conditions (Q1), at an intermediate level at elevated discharge (Q2), and highest at high discharge (Q3; Supplementary Figure 1).

### Stable Taxonomic Composition of Dominant Community Members

Bacterial communities in all samples were dominated by four different phyla and candidate phyla that consistently contributed between 66.3 and 83.6% (mean = 75.8%) of all reads over the entire sampling period of more than 27 month (Supplementary Table 1A). These included (1) candidate phylum OD1 (also referred to as Parcubacteria; Rinke et al., 2013), (2) phylum *Proteobacteri*a, (3) candidate phylum GN02 (also known as candidate phylum BD1-5 and renamed to Gracilibacteria) as well as (4) the phylum *Bacteroidetes*. Figure 3 illustrates the taxonomic composition of bacterial communities on class level for all samples grouped in classes of prevailing discharge conditions during sampling. In all three discharge groups (Q1–Q3), the seven bacterial classes with highest relative abundance in all samples contributed at least 53.2% of all sequence reads (Supplementary Table 1B). Interestingly, the 20 most abundant bacterial classes are identical in either baseflow or event-spring water samples, and their ranks in relative abundance shift only slightly between the two sample groups (Supplementary Table 2A). Moreover, out of the six classes with highest relative abundance (excluding “*All other* bacteria”) in either discharge group, four do not have any cultivated representative to date. Besides, only one sample, namely BF10, was identified as clear outlier based on taxonomic composition. In that sample, *Alphaproteobacteria* where clearly increased in relative abundance. Further analyses revealed an affiliation to the genus *Nitrobacter* with a relative read abundance of 22.6% in that sample. Supplementary Figure 3 depicts the taxonomic composition of the bacterial communities in all samples on highest possible taxonomic resolution down to the genus level.

### Community Variability Over Time

**FIGURE 4:**
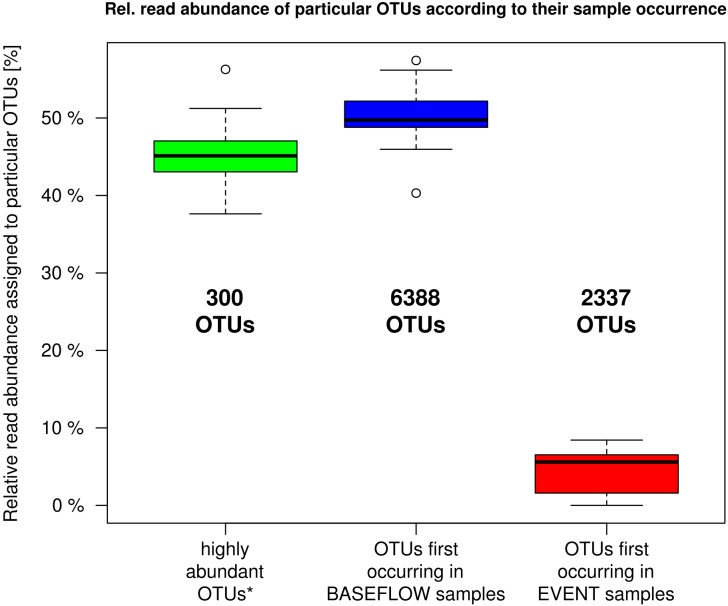
Relative read proportion assigned to OTUs categorized based on their first occurrence in any one sample in chronological order (cf. Supplementary Figure 2). Left boxplot: most abundant OTUs that showed > 100 reads per sample and appeared in at least 27 out of 32 samples (*n* = 300); Center boxplot: less abundant OTUs that showed their first occurrence in a baseflow-sample (BF01–BF16); Right boxplot: less abundant OTUs that first occurred in an event sample (EV01–EV16).

The analysis of the number of OTUs occurring for the first time in any one sample sorted in chronological order revealed a rapid decline and saturation of new OTUs (Supplementary Figure 2). OTU-based community analysis further revealed an abundant core community of 300 OTUs. These “*core OTUs*” on average contributed 45% (38–56%) of all sequence reads in each sample (Figure 4). In contrast, a considerably larger number of 6388 low abundant OTUs contributed a similar mean portion of 50% (40–57%) of all reads per sample (Figure 4).

During the investigated high-discharge event, including 16 samples collected over ∼2 weeks, 2337 new OTUs were identified that have not been detected in any of the baseflow samples collected prior to the event (Figure 4). These OTUs on average contributed only 4.4% (0–8.4%) of all reads in any one sample. Marked proportional changes in relative read abundance during the event were recorded in particular for Parcubacteria (OD1), which increased by almost 50% in median relative abundance in event samples when compared to baseflow conditions (cf. Supplementary Table 2A and Figure 3). On the other hand, sequences assigned to *Betaproteobacteria* (to a major part ascribed to the genus *Rhodoferax*) as well as *Nitrospira*-affiliated reads showed pronounced decreases in relative abundance during the event (Figure 3 and Supplementary Table 2A). Yet, the most marked change on taxonomic level was observed for the class *Flavobacteriia* (phylum *Bacteroidetes*), for which the ratio of the mean relative abundance of sequences between EV samples and BF samples was larger than 2 (Supplementary Table 3). While on average comprising only 2.3% of all reads under baseflow conditions, they contributed more than 12.7% of all sequence reads at a maximum in the event samples (Supplementary Table 2A).

### *Flavobacteriia* as Most Responsive Taxonomic Group During a High Discharge Event

**FIGURE 5:**
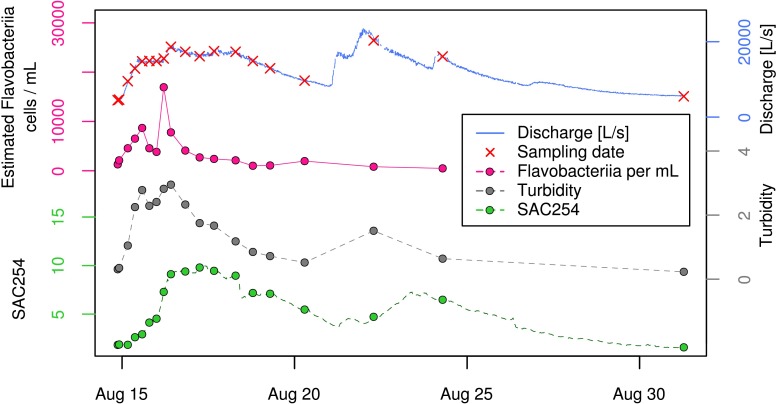
Time-course of “estimated *Flavobacteriia* concentrations” (pink) along with physical and chemical parameters during the investigated high-discharge event. Spectral Absorption Coefficient at λ = 254 nm (SAC_254_).

To put shifts in relative proportion into context of total bacterial dynamics of this hydrologically dynamic aquifer, we estimated absolute cell concentrations (from here on referred to as “estimated cell/clade-concentrations”) based on clade proportions and total cell counts (see section “Materials and Methods” and Supplementary Table 2B). While the ratios of mean estimated cell-concentrations between EV and BF samples were between 3 and 3.7 for three out of the four most abundant bacterial classes (i.e., “uncultured bacterium [Parcubacteria (OD1)],” “*Alphaproteobacteria* [*Proteobacteria*],” and “uncultured bacterium [Gracilibacteria (GN02)]”), *Flavobacteriia*-affiliated reads showed a ratio of 5.5 between the two sample types (Supplementary Table 2B). While not even ranked within the 10 most abundant classes under baseflow conditions, this group was the fifth most abundant class during the event (Supplementary Table 2B). Thereby, their increase was mainly restricted to the early phase of the studied high-discharge event, with two consecutive peaks and an intermediate drop in estimated absolute cell concentrations within ∼15 h (Figure 5). When calculating the ratio between the maximum estimated *Flavobacteriia* concentration during the event (18,920 cells mL^−1^; sample EV06) and the mean estimated cell concentration under baseflow conditions (988 cells mL^−1^), this corresponds to an almost 20-fold increase (Supplementary Table 3). In contrast, the respective ratios for the two most abundant bacterial groups (“uncultured bacterium [Parcubacteria (OD1)]” and “*Alphaproteobacteria* [*Proteobacteria*]”) were at 7.9 and 7.7, respectively (Supplementary Table 3). In context of hydrogeology, close resemblance could be observed between the observed patterns in estimated absolute *Flavobacteriia* concentrations, discharge, and turbidity, in particular during the early phase of the event (Figure 5). In contrast, SAC_254_ as a measure for dissolved organic compounds peaked with clear delay to discharge and remained at elevated levels for more than 10 days (Figure 5). SFIB concentrations such as *E. coli*, enterococci and the ruminant-associated genetic fecal MST-marker BacR showed a clear increase at the beginning of the event (shown for *E. coli* in Figure 1B). In a second high-discharge peak occurring exactly 1 week later (Aug 22; Figure 5), however, concentrations of *Flavobacteriia*, *E. coli* as well as for enterococci did not show an increase as observed in the first event, while turbidity and SAC_254_ did indeed increase again.

The detailed analysis of the two consecutive peaks of elevated estimated absolute *Flavobacteriia* concentrations revealed that these were caused by two distinct *Flavobacteriia*-OTUs, namely OTU_6 and OTU_7. While OTU_7 was the main contributor to the first peak, OTU_6 signed responsible for the second one. The alignment of the representative sequences revealed a sequence similarity of 96% (237/247 bp) for the partial 16S rRNA gene sequences, including a 14 bp short region containing 5 mismatches. Both sequences were assigned to the genus *Flavobacterium* but could not be further classified.

## Discussion

Still today, there is only a limited number of available studies investigating the bacterial community composition in spring water of karst aquifers – despite their huge importance as drinking water resources all around the world. While several studies investigated the bacterial communities of karst aquifers with temporally severe impact from surface-environments (Pronk et al., 2006, 2009), to our knowledge, only one study focused on the bulk bacterial community structure in an *alpine* karst spring (Farnleitner et al., 2005).

### Abundant Bacterial Populations in the Baseflow Component in an Alpine Spring Water

The observation of a stable suspended bacterial community consistently prevailing in the studied aquifer with a catchment of ∼70 km^2^ over the entire sampling period of more than 27 months supports findings from previous studies (Farnleitner et al., 2005; Pronk et al., 2006). Interestingly, two out of the three most abundant bacterial phyla, namely candidate phyla Parcubacteria (OD1) and Gracilibacteria (GN02), still today do not have any cultivated representative. In contrast, representatives of two other abundant phyla, *Proteobacteria* and *Bacteroidetes*, are well-known to comprise many typical freshwater bacteria and have been reported from most aquatic ecosystems (Zwart et al., 2002; Newton et al., 2011). The high abundance of Parcubacteria (OD1) as well as Gracilibacteria (GN02) was of further interest because previous sequencing-based studies on similar systems did not report a comparable representation of these candidate phyla (Farnleitner et al., 2005; Pronk et al., 2009). To validate these differences, a re-analysis of previously deposited sequences of these studies was conducted using the SINA online-aligner (Pruesse et al., 2012) and latest SILVA sequence-database (SSU v128; Yilmaz et al., 2014), allowing only 3 out of 134 sequences (∼2%) reported by Pronk et al. (2009) to be assigned to the phylum Parcubacteria (OD1). Yet, a very recent study on the bacterial communities in deep terrestrial subsurface aquifers of the Fennoscandian Shield in Sweden revealed comparably high abundances of Parcubacteria (OD1), with highest proportions at a sampling site 290 m below the surface, featuring a hydrological retention time of ∼5 years (Hubalek et al., 2016). This congruency was of particular interest since the studied aquifer in that study is located in a granite and quartz-monzodiorite-composed geologic formation which is highly dissimilar to the karst aquifer studied in the present study. This observation might in turn suggest a general importance of bacteria affiliated to this candidate phylum in deep cold groundwater aquifers, which, until recently, has rather often been reported from a broad range of mainly anoxic environments (Harris et al., 2004; Peura et al., 2012; Nelson and Stegen, 2015). However, a very recent study could also recover eight partial to near-complete genomes from an oxic groundwater environment (Nelson and Stegen, 2015). The available information on their physiology based on these metagenomic and single cell sequencing studies suggests generally small genomes with severely reduced metabolic capabilities as well as a potential ectosymbiotic or parasitic lifestyle (Kantor et al., 2013; Rinke et al., 2013; Wrighton et al., 2014; Brown et al., 2015; Nelson and Stegen, 2015; Castelle et al., 2017, 2018; Castelle and Banfield, 2018).

Regarding the other abundant phyla, sequences assigned to the genus *Rhodoferax* (family *Commamonadaceae*) of the class *Betaproteobacteria* contributed an average proportion of 3.3% of all reads during baseflow conditions, and have previously been suggested to play an important role in karstic aquatic systems (Shabarova et al., 2013). Besides, also the genus *Leptospirillum*, affiliated to the phylum *Nitrospirae*, contributed an average proportion of 2.61% of all sequence reads during baseflow conditions. The presence of bacteria affiliated to the phylum *Nitrospira* has previously also been reported from several other karst systems (Farnleitner et al., 2005; Pronk et al., 2009; Kostanjšek et al., 2013).

### Speculations on the Potential Lifestyle of the observed Alpine Karst Communities

The combination of (i) relatively low water retention times, (ii) low availability of organic nutrients, and (iii) low water temperatures as characteristic for many *alpine* karst aquifers in particular during baseflow conditions opposes the idea of prosperous heterotrophic growth and stable symbioses within the water column. Consequently, the high abundance of supposedly symbiotic or parasitic bacteria as previously suggested for Parcubacteria (OD1) (Castelle et al., 2018 and papers within) in the water column even during baseflow conditions may suggest an origin from sessile biofilms and a continuous detachment of cells e.g., from conduit walls or sediments (Savio et al., 2018). A high importance of biofilm-associated microbial activity has already previously been proposed in a study investigating 12 *alpine* karst aquifers (including also LKAS2), revealing pronouncedly higher heterotrophic prokaryotic production rates in sediments when compared to the bacteria suspended in the water column (Wilhartitz et al., 2009). In this context, an increasing number of studies is challenging the traditional view of subsurface environments’ dependency on surface-derived, allochthonous carbon inputs, but rather suggest a high importance of chemolithoautotrophic metabolic processes such as the utilization of reduced sulfur and nitrogen compounds as energy sources as recently shown for a shallow fractured carbonte-rock model aquifer in central Germany (Herrmann et al., 2015, 2017; Kumar et al., 2017, 2018; Starke et al., 2017). Correspondingly, also a recent study on bacterial biofilm-formations attached to the stream bed of a dinaric karst cave in Slovenia reported a high abundance of bacteria affiliated to the phylum *Nitrospirae*, well known for their chemolithoautotrophic lifestyle (Kostanjšek et al., 2013). Yet, the presented data from the present study do not provide sufficient evidence to draw final conclusions about the primary habitat and probable lifestyle of the bacteria observed in the water column. For example, high proportions of Parcubacteria (OD1) have previously also been reported from alpine permafrost soil (Frey et al., 2016). Therefore, future studies will have to shed light on the pending question of a proposed biofilm-associated lifestyle and origin from within the aquifer, demanding either for time-intensive *in situ* growth experiments (c.f. Savio et al., 2018), or cave diving expeditions. In addition, future studies should also address the question for the controlling agents in the microbial food-web of *alpine* karst aquifers. In this regard, a previous study proposed negligible grazing pressure by eukaryotes (heterotrophic nanoflagellates) but main control by nutrient availability and viral lyses based on a comparison of prokaryote-to-heterotrophic nanoflagellate and virus-to-prokaryote ratios in *alpine* karst systems with those of other aquatic systems (Wilhartitz et al., 2013).

### Detectable Population Variability Under Increased Discharge and Surface Influence

The major limitation of the few available DGGE-based studies on the bacterial community composition in spring water of *alpine* karst aquifers was the methodological restriction to detect the dynamics of low abundant populations below 1–10% in relative abundance (Farnleitner et al., 2005; Pronk et al., 2006). The present study based on high-resolution community fingerprinting using high-throughput 16S rRNA gene amplicon sequencing was able to prove a clear impact of the prevailing discharge conditions on the suspended bacterial community structure. Thereby, the discharge-induced changes were clearly reflected on multiple levels, including *alpha* and *beta diversity* as well as taxonomic community composition. Bacterial community dissimilarities as revealed by *beta diversity* analysis in combination with hydrological background data allowed the definition of three different discharge classes (Q1–Q3) for the studied spring. The observation of an increasing species richness during the studied high-discharge event might indicate the direct import of allochthonous bacteria from surface-environments. Although such surface-derived origin could not be proven in the present study, clear evidence for a direct impact from surface-associated habitats was indicated by feces-associated indicator bacteria such as *E. coli*, enterococci and a genetic *Bacteroidetes*-specific MST marker, as well as by copiotrophic indicators HPC22. All these parameters have previously been proven to represent reliable indicators of surface influence (Pronk et al., 2007, 2009; Reischer et al., 2008; Farnleitner et al., 2010; Sinreich et al., 2014). In the present study, these indicators increased by factors between ∼100 and 506 when calculating the ratio between maximum concentrations during the event and mean concentrations during baseflow conditions (Supplementary Table 3).

In contrast, the corresponding ratio for the most responsive group according to taxonomic analysis, the bacterial class of *Flavobacteriia*, was at ∼20. However, besides a generally much higher absolute abundance when compared to classical indicators (e.g., by a factor of ∼10 000 for mean concentrations of *Flavobacteriia* and *E. coli* during the event; cf. Supplementary Table 2B), this bacterial group might be of general relevance as indicator for the impact of high-discharge events in *alpine* karst aquifers. In this regard, also a previous study on the planktonic bacterial communities in stagnant rock pools reported elevated *Flavobacteriia* concentrations in the aftermath of several high-discharge events, and suggested a possible connection between specific flavobacterial populations and major surface-related events (Shabarova et al., 2014).The results of the present study clearly support such an association with high-discharge events. However, despite indicating the impact of a high-discharge event, these bacteria must not necessarily be of allochthonous, surface-derived origin. Our data rather suggest that the two most abundant *Flavobacteriia*-affiliated OTUs are members of a stable autochthonous spring water community (“AMEC,” cf. Farnleitner et al., 2005; Savio et al., 2018). This perception is based on two arguments: (1) *Flavobacteriia* consistently contribute to the spring water community also during baseflow-conditions and in the absence of detectable precipitation-induced impacts from surface habitats, and (2) their estimated absolute cell concentrations in spring water show close association with temporal dynamics in turbidity (Figure 5) – a well-accepted indicator for the mobilization of system-internal, alluviated inorganic sediments in *alpine* karst systems during the very early phase of a high-discharge event (Pronk et al., 2006; Stadler et al., 2008, 2010). In contrast, values for SAC_254_ – a well-known and commonly applied indicator for allochthonous, most likely surface-derived dissolved organic input (e.g., from soils) in karst systems (Stadler et al., 2010) – increased with clear delay when compared to turbidity and *Flavobacteriia* concentrations. An internal but not soil-associated primary origin of the observed responsive *Flavobacteriia* was further supported by blasting the representative sequences of the two dominant OTUs (OTU_6 and OTU_7) against 266,675 publicly available 16S rRNA gene libraries (of the V1–V2 variable region of the 16S rRNA gene) using the IMNGS-pipeline (Lagkouvardos et al., 2016). This pipeline gives insights about the most probable habitat a 16S rRNA gene sequence originates from based on the habitat-distribution of its closest relative sequences deposited in publicly available short read archives (SRA). Analyzing the two most abundant flavobacterial sequences, the analysis revealed markedly higher probabilities for a freshwater-associated (“freshwater metagenome”) than a soil-derived (“soil metagenome”) primary origin (Supplementary Table 4). Yet, a possible deposition and subsequent persistence of surface-derived flavobacterial cells in the aquifer’s sediments after an earlier high-discharge event in combination with a “cool storage”-effect in these cold subsurface environments cannot be entirely excluded. However, results from Shabarova et al. (2013) on the succession of planktonic cells in stagnant karst rock pools after the occurrence of a flood suggested rather the opposite based on relatively high dynamics of Flavobacteria-OTUs. In general, members of the genus *Flavobacterium* are well known to commonly inhabit a wide range of temperate and cold soil and water habitats, and to play an important role in biofilm-formation based on the gliding motility of many of its members, which is expected to facilitate the colonization of surfaces (Kirchman, 2002; Cousin et al., 2009; Battin et al., 2016). A preferential mobilization from biofilms could for example arise from the biofilm-structure itself, potentially exposing in particular *Flavobacteriia* due to their localization near the biofilm surface. Although the question for their primary origin and functional role remains unanswered, our observation from a single high-discharge event suggest that these bacteria may have indicator potential for mobilization processes within the karst-aquifer, and that they could therefore serve as early warning indicators for approaching water quality-deteriorations.

### Potential Microdiversity Among *Flavobacteriia* and Its Potential Future Utility for Water Quality Monitoring

The observation that two different *Flavobacterium*-affiliated OTUs dominated either of the two consecutive *Flavobacteriia*-peaks during the high-discharge event may indicate a possible micro-diversification of sublineages within the widely branched subsystems of the large karst aquifer system, draining a catchment of ∼70 km^2^. Such microdiversification might be a result of adaptation to distinct ecological niches due to differential oxygen or nutrient availability provided within the karst aquifer. If this hypothesis of subcatchment-specific sublineages, species or strains can be confirmed in the future, they may harbor great potential as natural indicators in biomonitoring (Pronk et al., 2009; Segawa et al., 2015). By specifically detecting and linking particular sublineages to locally isolated precipitation events in the catchment, their (increased) abundance may give information about preferential flow paths and retention times from different subcatchments. From a technical point of view, the observed higher concentrations of *Flavobacteriia* compared to cultivation-based indicators such as HPC counts harbor the potential for near-real time detection by optical (FISH in combination with Flow Cytometry) or molecular biological (qPCR, ddPCR) detection methods to complement time-intensive, but highly sensitive cultivation-based methods for the detection of SFIBs. This, however, requires further advancements in particular regarding the automated on-site sample-processing such as for the specific labeling of target cells (e.g., by FISH-techniques) for on-site flow cytometry applications, which already today are commonly applied to investigate microbial dynamics with unique temporal resolution (Besmer et al., 2016; Page et al., 2017; Van Nevel et al., 2017). Before these methods will be available, future studies should combine near real-time Flow Cytometry for TCC determination combined with biologically replicated sampling for DNA-based community analyses to also address the question of short-term dynamics within the community.

## Conclusion

Applying high-throughput 16S rRNA gene amplicon sequencing to a hydrogeologically defined set of baseflow and event samples, we observed a taxonomically stable bacterial community prevailing over more than 2 years in the studied *alpine* karst aquifer. The high contribution of so far uncultivated phyla provides room for speculations and allows to formulate research hypotheses to address questions related to their primary origin and potential functional role in the aquifer. Apart from a generally high stability in the bacterial community structure, we could also detect clear response in the abundance of particular bacterial groups to changing discharge-conditions especially during a studied high-discharge event. In this context, bacteria affiliated to the class of *Flavobacteriia* showed highest response and might harbor potential as a valuable natural indicator for “system disturbance” in karst aquifers.

## Author Contributions

DS, AF, AB, HS, and RM contributed conception and design of the study. DS designed and performed the laboratory experiments/work and analyzed the data. PS organized and analyzed physical and chemical data and helped with hydrological sample characterization and data interpretation. GR helped with sampling, method consulting, laboratory work, on-site physical and chemical measurements, data interpretation, and manuscript drafting. KD and RL helped with data analysis and data visualization. AB and HS helped with hydrogeological data interpretation. AK conducted optical measurements and helped with data analysis and manuscript drafting. AF together with DS wrote the first draft of the manuscript. All authors contributed to manuscript revision, read, and approved the manuscript.

## Conflict of Interest Statement

The authors declare that the research was conducted in the absence of any commercial or financial relationships that could be construed as a potential conflict of interest.
