# Supplementary material for: Spring Water of an Alpine Karst Aquifer Is Dominated by a Taxonomically Stable but Discharge-Responsive Bacterial Community
Source: Front Microbiol. 2019 Feb 15;10:28. doi: 10.3389/fmicb.2019.00028 (PMC6385617; doi:10.3389/fmicb.2019.00028)
Supplement: Supplementary file 1 [file Data_Sheet_1.docx]

Supplementary Material

Spring water of an alpine karst aquifer is dominated by a taxonomically stable but discharge-responsive bacterial community

**Domenico Savio^*^, Philipp Stadler, Georg H. Reischer, Katalin Demeter, Rita B. Linke, Alfred P. Blaschke, Robert L. Mach, Alexander K.T. Kirschner, †Hermann Stadler, Andreas H. Farnleitner^*^**

*** Correspondence:** Andreas Farnleitner: [andreas.farnleitner@kl.ac.at](mailto:andreas.farnleitner@kl.ac.at)

Domenico Savio: [domenico.savio@kl.ac.at](mailto:domenico.savio@kl.ac.at)

# Supplementary Data

## Supplementary Material & Methods

**Detailed description of the used two-step barcoding procedure for the preparation of 16S rRNA gene libraries**

In a first step, the V1-V2 hypervariable regions of bacterial 16S rRNA genes were amplified in triplicate PCR reactions containing 2.5 µL 10x reaction buffer (Easy-a cloning enzyme buffer; Agilent Technologies, Inc., US), 1 mM dNTPs, 0.625 units Easy-a cloning enzyme (Agilent Technologies, Inc., US), 0.2 µM of primer 8F (5’-AGAGTTTGATCCTGGCTCAG-3’; S-D-Bact-0008-a-S-20; Frank et al., 2007) and 338 (5’-TGCTGCCTCCCGTAGGAGT-3’; S-D-Bact-0338-a-A-19; Fierer et al., 2008), 5 µL diluted environmental DNA as template, and pure water (Merck) summing up to a final volume of 25 µL per reaction. PCR was performed with an initial denaturation step of 2 min, 25-30 cycles of 30 sec denaturation at 94 °C, 45 sec annealing at 57 °C and 1 min elongation at 72 °C, and a final extension step of 2 min. General cycle number was set to 25 with one exception for sample BF10, for which 30 cycles of amplification were conducted to retrieve a sufficiently high amplicon-concentration for later use in the second step PCR. Dilution factor of environmental DNA used in the 1st step PCR was determined based on 16S rRNA gene copy number measurements by 16S rRNA gene specific quantitative PCR (see Material & Methods section in main text). In case of PCR-inhibition as a result of co-extracted inhibitory substances, a higher dilution of environmental DNA was used. Based on these preconditions, 5 µL of undiluted environmental DNA were used for samples BF04, BF10, BF11 and BF13, 5 µL of 1:4.5 diluted DNA solution were used for samples BF01, BF02, BF05, BF06, BF07, BF12, BF14, BF15 and EV01, and 1:20.25 diluted DNA was used for samples BF03, BF12 and BF16. Highly concentrated or inhibited samples were samples BF08, BF09 and EV02-EV16, for which 1:64 diluted DNA was used. For samples EV06, EV12 and EV14, 1:256 diluted DNA was used.

In a second step, amplicons of triplicate reactions from 1st step PCR were pooled and 0.5 µL of amplicon solution used as template DNA in a 2nd step re-amplification PCR – again prepared in triplicates – with few adaptations to the above described protocol. Most important was the use of modified versions of the reverse primer instead of classical reverse primer 338, containing unique 12bp long multiple-identifier (MID)-sequences (commonly also referred to as “barcodes”) as well as Roche Adapter “Primer A” (5’-GCCTCCCTCGCGCCATCAG-‘12bp barcode sequence’-3’; see Supplementary Table 5) at the 5’-end of primer 338 (Fierer et al., 2008). Unique ‘barcode sequences’ were introduced for each sample which allowed the demultiplexing of sequences from different samples after High-Throughput-Sequencing (HTS). Forward primer 8F (Frank et al., 2007) was also extended at its 5’-end with Roche Adapter “Primer B” (5‘-GCCTTGCCAGCCCGCTCAG-3’). Sample-specific “barcoding” during this 2nd step PCR was conducted in only 8 cycles to reduce possible PCR-biases due to long overhangs (Berry et al., 2011).

After purification of amplicons from pooled triplicate reactions with the Mag-Bind® E-Z Pure purification kit (Omega Bio-Tek, USA) following the manufacturer’s instructions, DNA-concentration of amplicon solutions were quantified using Quant-iT PicoGreen dsDNA Assay Kit (Life Technologies Corporation, USA) on an Anthos Zenyth 3100 Microplate Multimode Detector and 100 ng of pure amplicon solutions were pooled together for each sample, resulting in a final measured concentration of the pooled sequence libraries of 5.44 ng µL^-1^.

**First occurrence analysis**

First-occurrence analysis was based on a self-written R-script and determines the number of novel OTUs occurring for the first time in every single sample. The output of the script is highly dependent on the initial sample-order in the OTU-Table. For the present study, samples were sorted in chronological order according to the date of sampling. The following lines describe the procedure of the used ‘first_occur’-function:

## First occurrence analysis script for the use in [R]

# IMPORTANT NOTE 1: The input for this script is an OTU-table with samples as rows and OTUs as columns.

# IMPORTANT NOTE 2: The samples must be in the correct order (row 1: sample 1, row 2: sample 2, row 3: sample 3, …) to get the desired output of first occurring OTUs in every single sample.

otutab <- OTU_tab_of_interest # assign OTU-Table (matrix or dataframe) of interest ‘OTU_tab_of_interest’ to 'otutab'

cd1 <- (otutab>0) # convert otutab to 1/0 (presence-absence) for each OTU in each sample

cd2 <- cd1*1 # convert TRUE/FALSE matrix to 1/0

cd3 <- apply(cd2, 2, cumsum) # cumulate all columns -> "1" appears the first time in those sample

cd4 <- (otutab==0) # create a matrix indicating all NON-occurrences of OTUs; no occurrence = 1, any occurrence = 0

cd5 <- cd4*max(colSums(cd3)) # convert to numeric from TRUE/FALSE by multiplication, and in order to avoid getting unwanted "1"s AFTER first "1" after first substraction (see below), MULTIPLY with max occurring number after cumulation -> would result in maximum of 0

cd6 <- (cd3-cd5) # remove 1's from positions where originally no counts were observed, but due to cumulation a 1 was written;

cd7 <- (cd6=="1")*1 # new matrix indicating the first occurrences, we are interested in the 1's = first occurrences

first_occur_OUTPUT <- rowSums(cd7) # assign sums of first occurrences to a vector

plot(first_occur_OUTPUT) # plot first occurrences

## Supplementary Information

**Detailed information regarding prevailing hydrological, physical and chemical conditions for the two sample groups and selected samples**

BF samples were taken at a mean discharge of 3,055 Ls^-1^ (1,257-5,513 Ls^-1^; n=14/16), while EV samples represented the bacterial communities at elevated discharge levels with a mean value of 15,213 Ls^-1^ (9,527-20,340 Ls^-1^; n=16; Table 1). No discharge data were available for two BF samples (BF04 & BF05). For sample BF04, no discharge data were recorded for almost 12 days before the sampling. However, values for turbidity as well as SAC_254_ suggested no prevailing surface-influence why this samples was categorized as BF sample. Samples BF02 as well as the ‘reference’ samples of the high-frequency sampling right before and after the studied high-discharge event (BF07-BF09) showed elevated SAC_254_ values either slightly above (BF02), or just below (BF07-09) the threshold value of 2. For sample BF02, a foregone high-discharge event was detected around 5 days before sampling.

**Further discussion of ‘outlier’ samples**

In the present analysis, sample BF10 was the only sample that appeared as clear outlier in beta-diversity as well as taxonomic analysis, and consequently could not be categorized according to the operationally defined classification system of 3 different discharge groups (Q1, Q2 and Q3). A technical explanation for this observation might be its divergent treatment during library preparation as described above (‘*Detailed description of the used two-step barcoding procedure for the preparation of 16S rRNA gene libraries*’), which was necessary due to extremely low DNA concentrations. A biological explanation might be the occasional input of soil-associated particles, which might be indicated by an unusually high proportion of ~22.56% of sequences affiliated to the genus *Nitrobacter*, well-known from most diverse soil habitats (Supplementary Figure 3).

Three additional samples assigned to discharge group 2 (namely BF07, BF08 and BF02) clustered notably closer to discharge group 3 samples in the NMDS-visualization of Bray-Curtis dissimilarities (Figure 2). This might be explained with the aftermaths of a previous (applying to sample BF02), or the already detectable influence of an arriving high-discharge event (samples BF07+BF08; see Supplementary Information above).

# Supplementary Figures and Tables

##
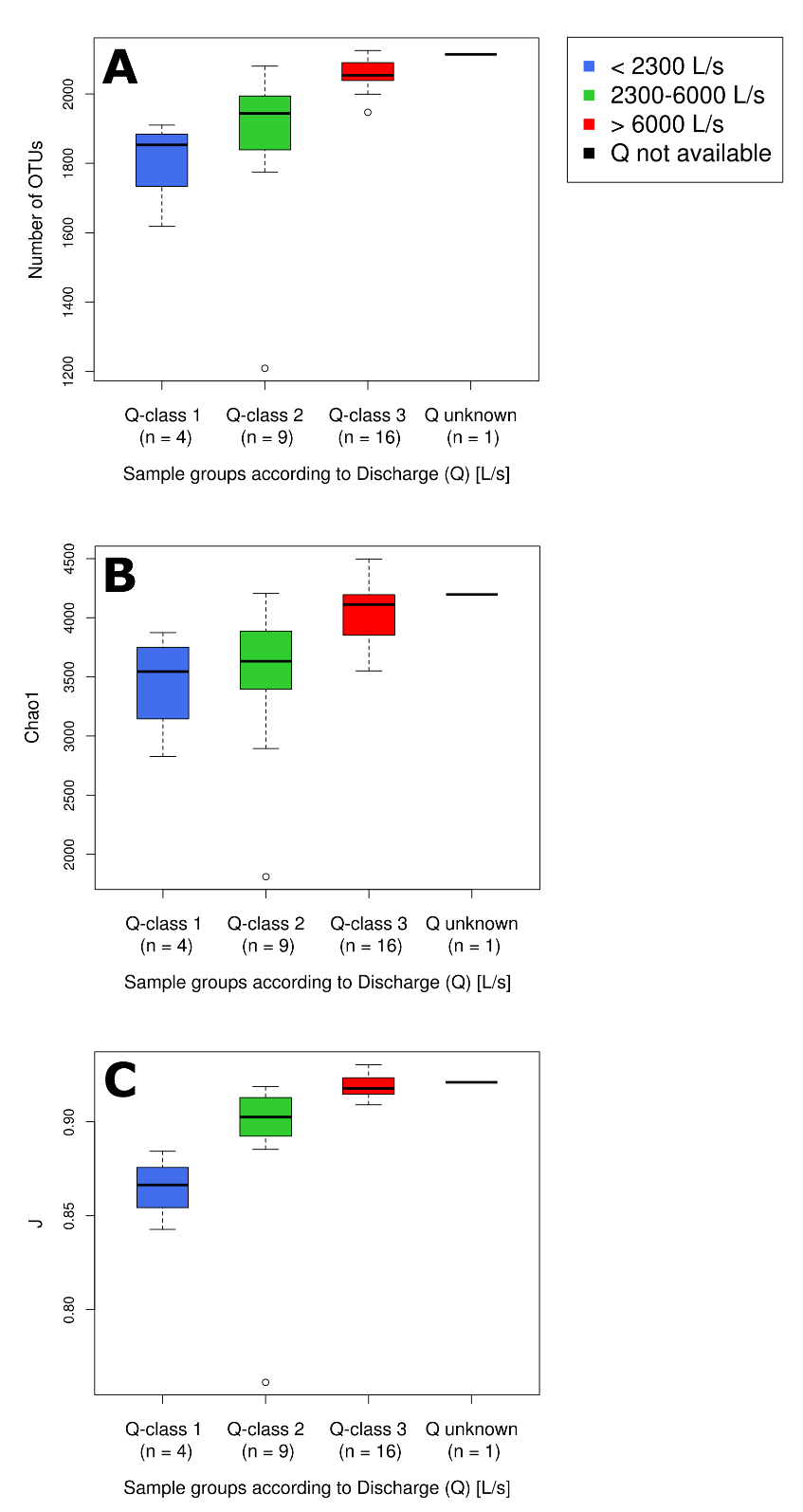
Supplementary Figures

**Supplementary Figure 1.** Box-plot-visualization of alpha diversity indices calculated for all studied samples with >5,116 reads (cf. Material & Methods) grouped by their respective discharge class based on the prevailing discharge at the time of sampling (cf. Figure 2). (A) shows the total number of observed OTUs, (B) displays Chao1 richness estimations, and (C) the Pielou’s Evenness (J).


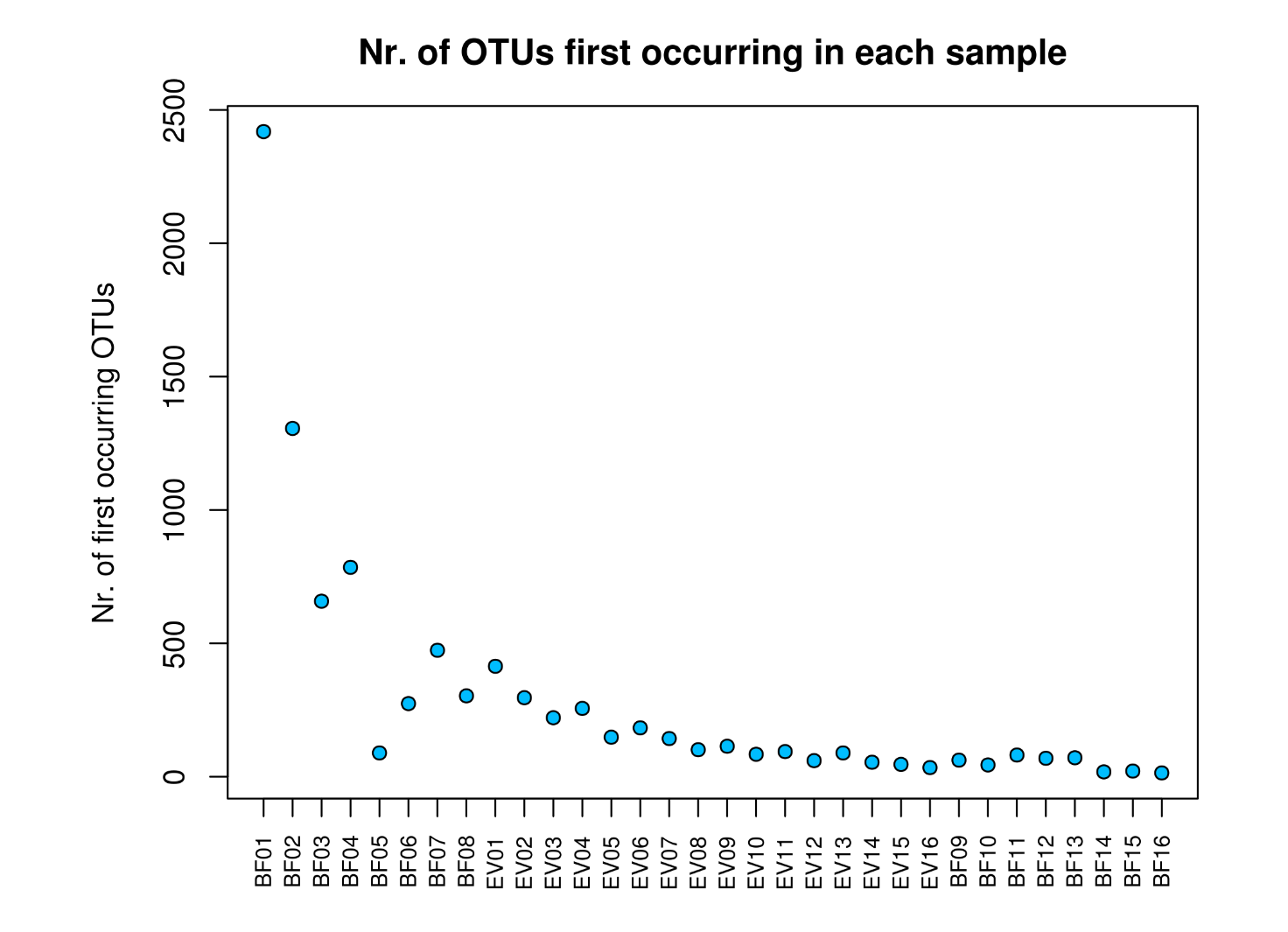


**Supplementary Figure 2.** Results from first occurrence analysis of OTUs: Y-axis depicts the absolute number of novel OTUs occurring in every single sample along the correct order of sampling, starting from left (BF01) to right (BF16), with high-discharge samples in between (EV01-EV16).

**
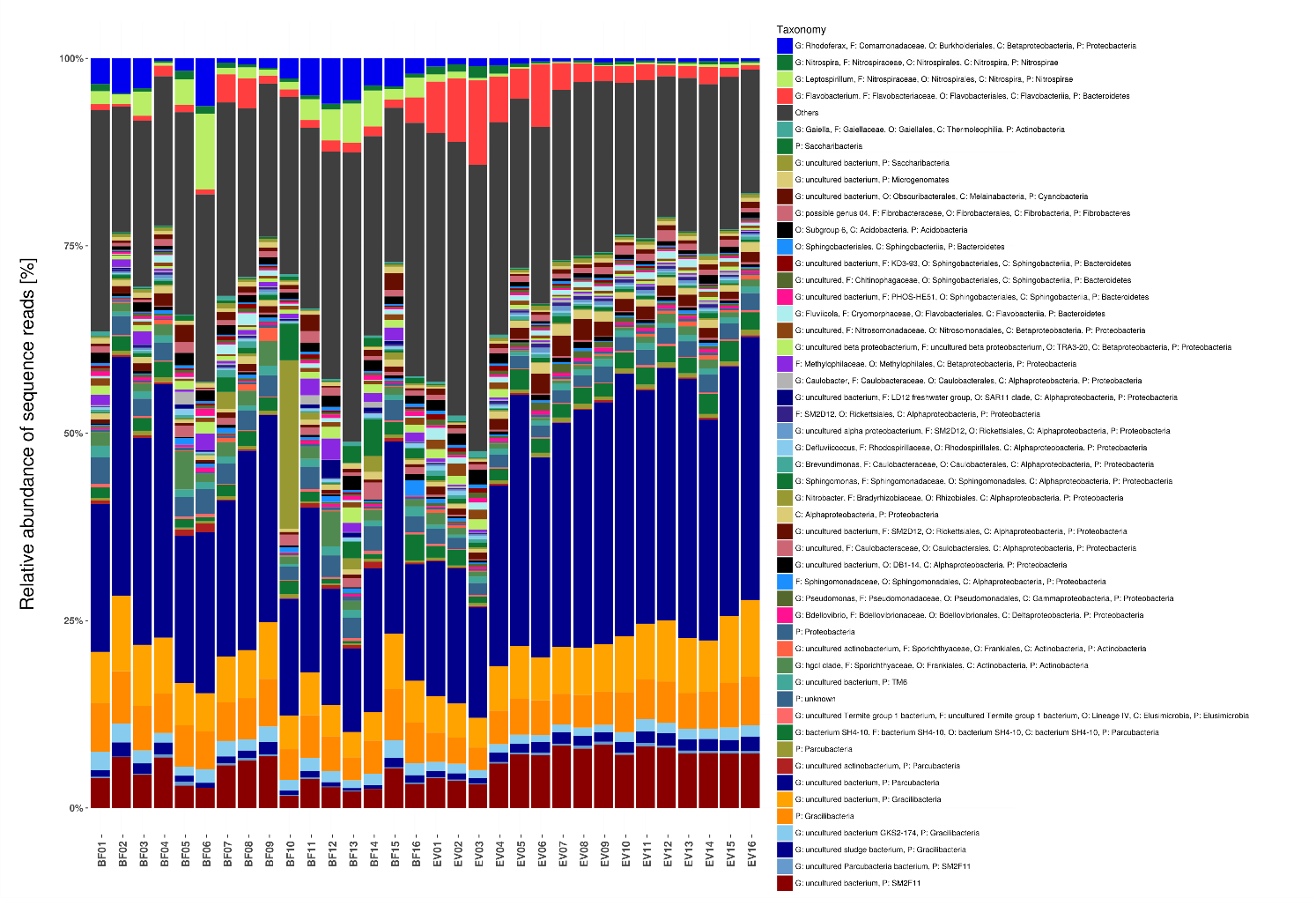
**

**Supplementary Figure 3.** Taxonomic community composition on highest possible taxonomic resolution down to the genus level for baseflow (left) and event samples (right), both sorted by time of sampling. Barstacks depict the relative abundance of the 50 most abundant bacterial classes according to SILVA taxonomy (v123; Yilmaz et al., 2014). All lower abundant groups were summarized within the group ‘Others’.


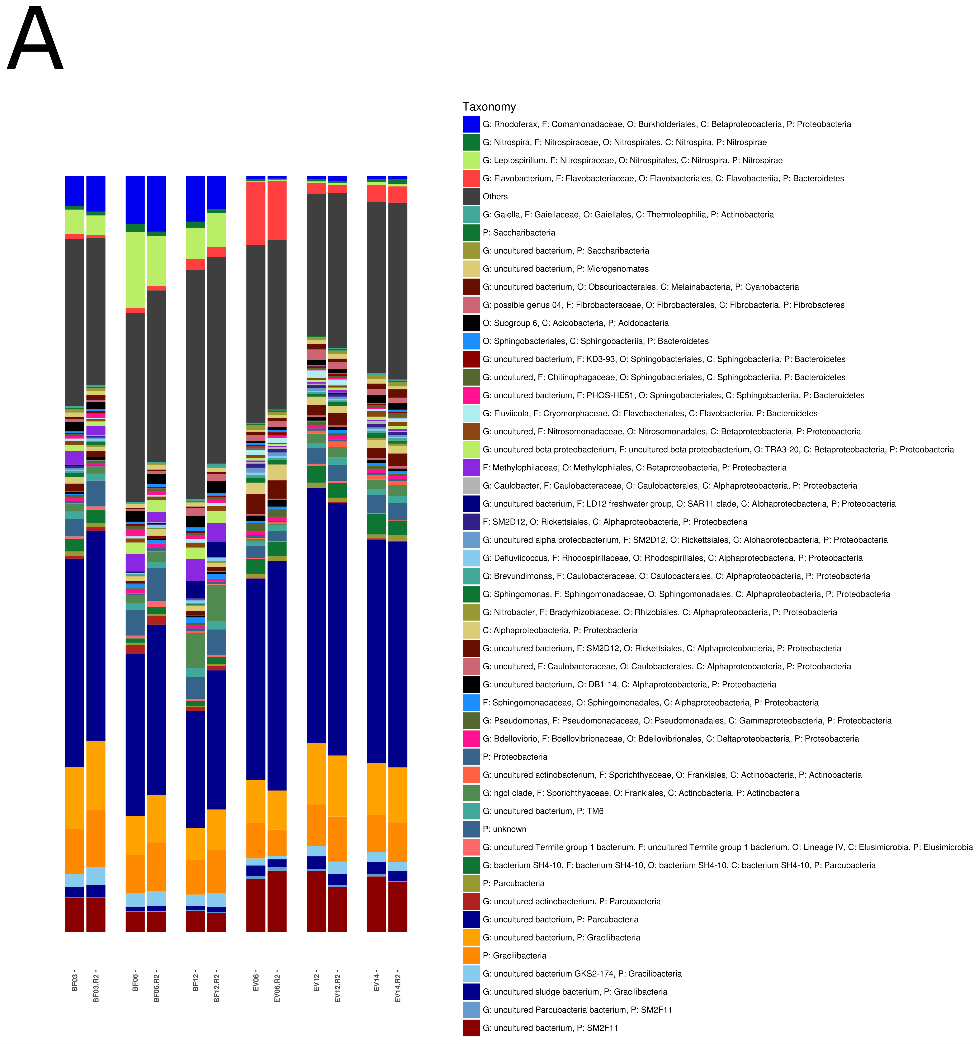


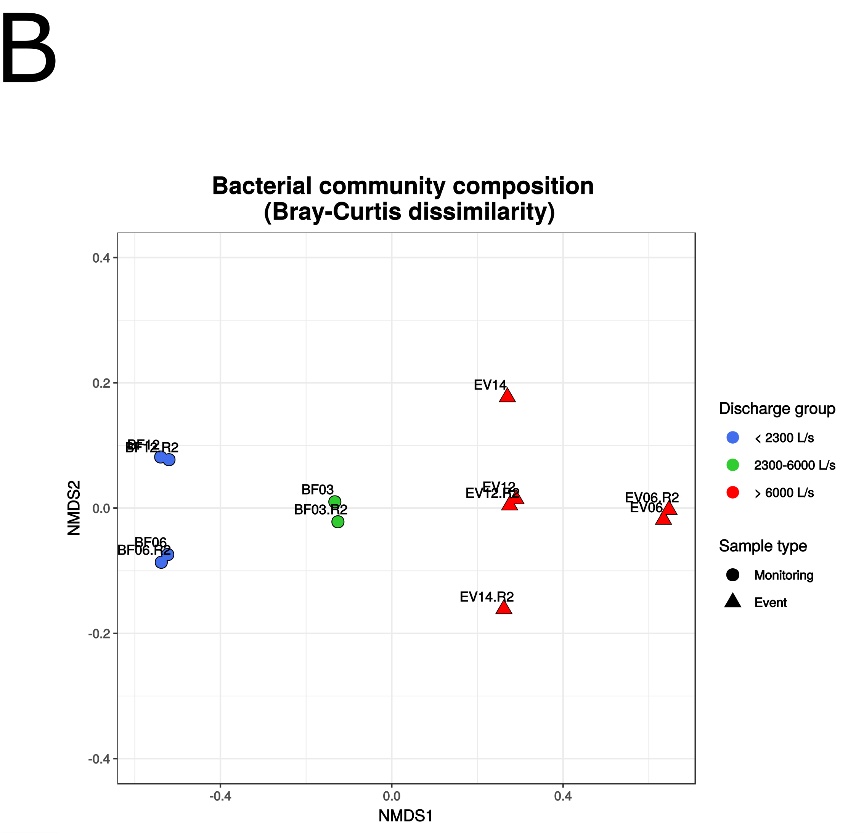


**Supplementary Figure 4.** (A) Taxonomic community composition on highest possible taxonomic resolution down to genus level for technical replicates of 6 selected samples (BF03, BF06, BF12, EV06, EV12, EV14 in that order). Replicate samples (extension ‘R2’) were identically treated during library preparation as conducted for the first replicates. Barstacks depict the relative abundance of the 50 most abundant bacterial classes according to SILVA taxonomy (v123; Yilmaz et al., 2014) and all lower abundant groups were summarized within the group ‘Others’. (B) NMDS-visualization of Bray-Curtis dissimilarities. ‘metaMDS’-function implemented in the R-package ‘vegan’ was used without allowance for prior autotransformation (Square-root and/or Wisconsin-Transformation) of data. Stress value of the NMDS was 0.0087.

## Supplementary Tables

**Supplementary Table 1.** Cumulative read proportions of the most abundant bacterial (candidate) phyla and classes according to Silva v123 taxonomy-assignment for different sample groups: (A) depicts the cumulative read proportions [%] for the 7 most abundant (candidate) phyla arranged in decreasing order by their relative abundance in all samples (BF+EV) for (i) all, (ii) baseflow and (iii) high-discharge event-samples. (B) shows the cumulative read proportions [%] for the 12 most abundant bacterial classes arranged in decreasing order by their relative abundance in all samples for (i) all samples as well as for (ii) discharge group 1 (Q1), (iii) discharge group 2 (Q2), (iv) discharge group 3 (Q3) and (v) those samples for which no discharge records were available (‘noQ’).

| **A** | | **ALL SAMPLES** | | | **BASEFLOW** | | | **EVENT** | | |  |  |  |  |  |  |
| --- | --- | --- | --- | --- | --- | --- | --- | --- | --- | --- | --- | --- | --- | --- | --- | --- |
| **(Candidate) Phylum** | | **Mean** | **Min** | **Max** | **Mean** | **Min** | **Max** | **Mean** | **Min** | **Max** |  |  |  |  |  |  |
| Parcubacteria (OD1) | | 28.3 | 12.2 | 38.6 | 24.7 | 12.2 | 37.0 | 31.8 | 16.3 | 38.6 |  |  |  |  |  |  |
| Proteobacteria | | 53.3 | 44.2 | 68.9 | 52.8 | 46.2 | 68.9 | 53.8 | 44.2 | 59.2 |  |  |  |  |  |  |
| Gracilibacteria (GN02) | | 68.1 | 53.4 | 80.0 | 67.8 | 60.0 | 80.0 | 68.4 | 53.4 | 75.7 |  |  |  |  |  |  |
| **Bacteroidetes** | | **75.8** | **66.3** | **83.6** | **74.0** | **66.3** | **83.6** | **77.5** | **74.0** | **80.8** |  |  |  |  |  |  |
| Doudnabacteria (SM2F11) | | 81.6 | 70.0 | 88.4 | 78.6 | 70.0 | 86.9 | 84.7 | 77.4 | 88.4 |  |  |  |  |  |  |
| Actinobacteria | | 85.1 | 76.6 | 89.9 | 82.8 | 76.6 | 89.3 | 87.3 | 81.8 | 89.9 |  |  |  |  |  |  |
| Nitrospirae | | 87.4 | 82.7 | 91.0 | 86.3 | 82.7 | 91.0 | 88.5 | 84.6 | 90.3 |  |  |  |  |  |  |
|  |  |  |  |  |  |  |  |  |  |  |  |  |  |  |  |  |
| **B** | | **ALL SAMPLES** | | | **Q1** | | | **Q2** | | | **Q3** | | | **noQ** | | |
| **(Candidate) Phylum** | **Class** | **Mean** | **Min** | **Max** | **Mean** | **Min** | **Max** | **Mean** | **Min** | **Max** | **Mean** | **Min** | **Max** | **Mean** | **Min** | **Max** |
| Parcubacteria (OD1) | uncultured bacterium | 25.5 | 11.2 | 35.1 | 18.1 | 11.2 | 22.6 | 23.4 | 15.5 | 31.8 | 28.8 | 14.7 | 35.1 | 26.7 | 19.6 | 33.8 |
| Proteobacteria | Alphaproteobacteria | 38.1 | 27.2 | 55.2 | 31.9 | 27.5 | 39.5 | 37.7 | 28.9 | 55.2 | 40.3 | 27.2 | 46.1 | 38.9 | 34.1 | 43.6 |
| Gracilibacteria (GN02) | uncultured bacterium | 44.6 | 30.9 | 59.7 | 36.6 | 30.9 | 43.3 | 44.6 | 35.7 | 59.7 | 47.0 | 31.1 | 54.6 | 45.4 | 39.7 | 51.1 |
| Proteobacteria | Betaproteobacteria | 50.9 | 38.7 | 65.8 | 48.5 | 45.6 | 51.8 | 51.6 | 44.9 | 65.8 | 51.5 | 38.7 | 57.2 | 49.5 | 44.0 | 55.0 |
| Doudnabacteria (SM2F11) | uncultured bacterium | 56.4 | 41.9 | 67.5 | 51.4 | 47.8 | 54.3 | 56.5 | 48.9 | 67.5 | 58.2 | 41.9 | 64.5 | 54.4 | 47.0 | 61.8 |
| Gracilibacteria (GN02) | Other | 61.5 | 44.8 | 72.6 | 56.2 | 50.8 | 59.1 | 62.3 | 54.6 | 72.6 | 62.9 | 44.8 | 70.9 | 59.8 | 52.5 | 67.1 |
| **Bacteroidetes** | **Flavobacteriia** | **65.2** | **53.2** | **73.3** | **58.0** | **53.2** | **61.0** | **65.0** | **56.9** | **73.3** | **68.0** | **57.5** | **72.2** | **61.7** | **53.8** | **69.7** |
| Bacteroidetes | Sphingobacteriia | 68.4 | 57.1 | 74.9 | 61.8 | 57.1 | 64.9 | 68.0 | 60.4 | 74.9 | 71.2 | 63.8 | 74.6 | 64.8 | 57.6 | 72.0 |
| Proteobacteria | Gammaproteobacteria | 71.2 | 59.3 | 78.2 | 65.3 | 62.5 | 67.8 | 70.4 | 63.2 | 78.2 | 74.0 | 67.6 | 78.2 | 66.8 | 59.3 | 74.4 |
| Proteobacteria | Deltaproteobacteria | 74.0 | 62.6 | 80.9 | 68.8 | 66.3 | 70.8 | 72.8 | 66.9 | 79.4 | 76.7 | 71.3 | 80.9 | 69.8 | 62.6 | 76.9 |
| Actinobacteria | Actinobacteria | 76.5 | 69.4 | 82.2 | 71.6 | 69.8 | 73.2 | 75.8 | 69.8 | 81.1 | 78.6 | 73.2 | 82.2 | 74.7 | 69.4 | 80.0 |
| Nitrospirae | Nitrospira | 78.8 | 73.2 | 82.8 | 77.3 | 76.3 | 79.1 | 78.2 | 73.2 | 82.8 | 79.8 | 76.0 | 82.6 | 77.7 | 74.6 | 80.8 |

**Supplementary Table 2. (A)** Relative proportions of 20 most abundant bacterial classes (Silva 123 taxonomy assignment) and all lower abundant classes summarized within the group ‘All other bacteria’ in decreasing order for baseflow samples (left), and high-discharge event samples (right). **(B)** Summary statistics of the estimated cell concentrations (cf. Material & Methods) for the ten most abundant bacterial classes in declining order of their estimated cell concentrations for baseflow (left) and event samples (right). Cell concentration estimates for each taxonomic group were calculated by multiplication of the relative read abundance of the respective class with the total concentration of prokaryotic cells per mL (TCC). SAC_254_, Spectral Absorption Coefficeint at 254 nm; FNU, Formazin Nephelometric Units; CFU, Colony Forming Units; HPC22 & HPC37, heterotrophic plate counts at 22 & 37 °C, respectively; BacR, & BacH, ruminant- and human-specific genetic fecal markers; ME, marker equivalents.

| **A** | **BASEFLOW** | | | | |  | **EVENT** | | | | | |
| --- | --- | --- | --- | --- | --- | --- | --- | --- | --- | --- | --- | --- |
|  | **n = 16** | **Mean [%]** | **Median [%]** | **Range (min-max) [%]** | **sd [%]** |  | **n=16** | **Mean [%]** | **Median [%]** | **Range (min-max) [%]** | **sd [%]** |  |
| **20 most abundant classes [%]** | **uncultured bacterium (Parcubacteria)** | 22.1 | 21.1 | 11.2-33.8 | 6.3 |  | **uncultured bacterium (Parcubacteria)** | 28.8 | 31.4 | 14.7-35.1 | 6.7 |  |
|  | **Alphaproteobacteria *(Proteobacteria*)** | 13.8 | 11.5 | 8.2-39.6 | 7.7 |  | **Alphaproteobacteria (*Proteobacteria*)** | 11.5 | 11.1 | 8.6-15.2 | 2.3 |  |
|  | **All other bacteria** | 11.3 | 11.0 | 8.3-14.5 | 1.7 |  | **All other bacteria** | 10.3 | 9.9 | 8.7-13.5 | 1.4 |  |
|  | Betaproteobacteria (*Proteobacteria*) | 8.1 | 8.5 | 3.6-14.7 | 3.5 |  | uncultured bacterium (SM2F11) | 6.8 | 7.3 | 3.2-8.5 | 1.7 |  |
|  | uncultured bacterium (Gracilibacteria) | 6.1 | 6.0 | 3.4-10.1 | 1.8 |  | uncultured bacterium (Gracilibacteria) | 6.7 | 6.6 | 4-10.3 | 1.6 |  |
|  | Other (Gracilibacteria) | 5.4 | 5.4 | 3.1-7 | 1.0 |  | **Flavobacteriia (*Bacteroidetes*)** | 5.1 | 3.4 | 1.2-12.7 | 3.3 |  |
|  | uncultured bacterium (SM2F11) | 4.3 | 4.0 | 1.6-6.9 | 1.8 |  | Other (Gracilibacteria) | 4.7 | 4.7 | 3-6.4 | 0.9 |  |
|  | **Nitrospira (*Nitrospirae*)** | 3.8 | 3.5 | 0.8-11.8 | 2.8 |  | Betaproteobacteria (*Proteobacteria*) | 4.5 | 3.9 | 2.6-7.5 | 1.6 |  |
|  | Sphingobacteriia (*Bacteroidetes*) | 3.3 | 3.8 | 1.4-5.2 | 1.0 |  | Sphingobacteriia (*Bacteroidetes*) | 3.1 | 2.7 | 1.6-6.3 | 1.4 |  |
|  | Actinobacteria (*Actinobacteria*) | 3.2 | 2.4 | 1.7-6.8 | 1.8 |  | Gammaproteobacteria *(Proteobacteria*) | 2.9 | 2.9 | 1.9-4.1 | 0.6 |  |
|  | Deltaproteobacteria (*Proteobacteria*) | 2.9 | 3.0 | 1.1-3.9 | 0.9 |  | Deltaproteobacteria (*Proteobacteria*) | 2.7 | 2.7 | 2-3.7 | 0.4 |  |
|  | Gammaproteobacteria (*Proteobacteria*) | 2.7 | 2.2 | 1.7-7.8 | 1.5 |  | bacterium SH4-10 (*Parcubacteria*) | 2.1 | 2.1 | 0.9-2.8 | 0.5 |  |
|  | **Flavobacteriia (*Bacteroidetes*)** | 2.3 | 1.9 | 0.6-5.9 | 1.5 |  | Actinobacteria (*Actinobacteria*) | 1.9 | 1.7 | 0.7-2.9 | 0.7 |  |
|  | Acidobacteria (*Acidobacteria*) | 2.3 | 2.3 | 1-4.2 | 0.8 |  | Acidobacteria (*Acidobacteria*) | 1.8 | 1.2 | 0.7-4.4 | 1.2 |  |
|  | uncultured bacterium GKS2-174 (Gracilibacteria) | 1.7 | 1.7 | 1-2.5 | 0.5 |  | uncultured sludge bacterium (Gracilibacteria) | 1.3 | 1.4 | 0.8-1.9 | 0.3 |  |
|  | bacterium SH4-10 (Parcubacteria) | 1.5 | 1.6 | 0.3-3.5 | 0.8 |  | uncultured bacterium GKS2-174 (Gracilibacteria) | 1.3 | 1.3 | 0.9-1.7 | 0.2 |  |
|  | Melainabacteria (*Cyanobacteria*) | 1.3 | 1.0 | 0.4-3.1 | 0.8 |  | **Nitrospira (*Nitrospirae*)** | 1.2 | 0.8 | 0.4-2.8 | 0.8 |  |
|  | Elusimicrobia (*Elusimicrobia*) | 1.1 | 1.1 | 0.5-1.9 | 0.4 |  | Fibrobacteria (*Fibrobacteres*) | 1.0 | 1.1 | 0.7-1.6 | 0.2 |  |
|  | uncultured sludge bacterium (Gracilibacteria) | 1.0 | 0.9 | 0.3-1.9 | 0.5 |  | Melainabacteria (*Cyanobacteria*) | 0.9 | 0.9 | 0.4-1.1 | 0.2 |  |
|  | uncultured bacterium (TM6) | 1.0 | 1.0 | 0.4-1.5 | 0.3 |  | Elusimicrobia (*Elusimicrobia*) | 0.8 | 0.9 | 0.5-1.1 | 0.2 |  |
|  | Fibrobacteria (*Fibrobacteres*) | 0.9 | 0.8 | 0.3-1.8 | 0.4 |  | uncultured bacterium (TM6) | 0.8 | 0.8 | 0.4-1 | 0.2 |  |

| **B** | **BASEFLOW** | | | | |  | **EVENT** | | | | |
| --- | --- | --- | --- | --- | --- | --- | --- | --- | --- | --- | --- |
|  | **n = 9** | **mean** | **median** | **Range (min-max)** | **sd** |  | **n = 16** | **mean** | **median** | **Range (min-max)** | **sd** |
| **Estimated cell conc. [x 10³ mL^-1^]** | **uncultured bacterium (Parcubacteria)** | 8.3 | 7.2 | 5.8-14.1 | 2.6 |  | **uncultured bacterium (*Parcubacteria*)** | 30.6 | 26.1 | 11.3-65.5 | 15.9 |
|  | **Alphaproteobacteria (*Proteobacteria*)** | 4.1 | 4.6 | 2.5-6 | 1.2 |  | **Alphaproteobacteria (*Proteobacteria*)** | 12.6 | 9.5 | 5.9-31.5 | 8.1 |
|  | Betaproteobacteria (*Proteobacteria*) | 2.8 | 3.1 | 1-5.3 | 1.5 |  | uncultured bacterium (SM2F11) | 7.4 | 5.9 | 2.5-18.2 | 4.5 |
|  | **uncultured bacterium (*Gracilibacteria*)** | 2.2 | 2.1 | 1.6-3.4 | 0.6 |  | **uncultured bacterium (*Gracilibacteria*)** | 6.9 | 6.4 | 3.1-13.7 | 3.0 |
|  | Other (Gracilibacteria) | 2.0 | 1.8 | 1.4-2.9 | 0.5 |  | **Flavobacteriia (*Bacteroidetes*)** | 5.4 | 4.3 | 1-18.9 | 4.5 |
|  | uncultured bacterium (SM2F11) | 1.6 | 1.6 | 0.8-3.3 | 0.8 |  | Other (Gracilibacteria) | 4.9 | 4.4 | 2.3-9.5 | 2.2 |
|  | **Nitrospira (*Nitrospirae*)** | 1.5 | 1.5 | 0.2-3.6 | 1.0 |  | Betaproteobacteria (*Proteobacteria*) | 4.5 | 4.5 | 2-8.9 | 2.0 |
|  | Actinobacteria (*Actinobacteria*) | 1.3 | 1.0 | 0.6-2.3 | 0.7 |  | Gammaproteobacteria (*Proteobacteria*) | 3.2 | 2.5 | 1.3-9 | 2.1 |
|  | Sphingobacteriia (*Bacteroidetes*) | 1.3 | 1.2 | 0.6-2 | 0.4 |  | Sphingobacteriia (*Bacteroidetes*) | 3.1 | 3.1 | 1.2-6.2 | 1.5 |
|  | Deltaproteobacteria (*Proteobacteria*) | 1.1 | 1.1 | 0.7-1.5 | 0.3 |  | Deltaproteobacteria (*Proteobacteria*) | 2.8 | 2.4 | 1.6-5.9 | 1.3 |
|  | **All lower abundant groups summarized** | 10.0 | 10.3 | 6.2-15.1 | 2.5 |  | **All lower abundant groups summarized** | 23.4 | 21.5 | 15.7-41.8 | 8.2 |
| **Surface indicators** (Reischer et al., 2008) | SAC_254_ [abs m^-1^] | 1.0 | 0.7 | 0.5-1.9 | 0.6 |  | SAC_254_ [abs m^-1^] | 6.3 | 6.8 | 1.8-9.8 | 2.7 |
|  | Turbidity [FNU] | 0.2 | 0.1 | 0-0.5 | 0.2 |  | Turbidity [FNU] | 1.7 | 1.7 | 0.5-3 | 0.8 |
|  | *E. coli* [CFU L^-1^] | 18.4 | 3.0 | 0-90 | 34.1 |  | *E. coli* [CFU L^-1^] | 521.6 | 275.0 | 45-1850 | 543.2 |
|  | Enterococci [CFU L^-1^] | 3.3 | 1.0 | 0-17 | 6.0 |  | Enterococci [CFU L^-1^] | 161.4 | 75.0 | 11-690 | 199.8 |
|  | Total Coliforms [CFU L^-1^] | 18.6 | 3.0 | 0-90 | 34.1 |  | Total Coliforms [CFU L^-1^] | 534.7 | 275.0 | 45-2060 | 578.8 |
|  | HPC22 [CFU mL^-1^] | 32.0 | 7.0 | 1-120 | 44.8 |  | HPC22 [CFU mL^-1^] | 1030.6 | 490.0 | 150-4600 | 1257.7 |
|  | HPC37 [CFU mL^-1^] | 3.6 | 0.0 | 0-19 | 6.8 |  | HPC37 [CFU mL^-1^] | 181.1 | 65.0 | 15-810 | 263.9 |
|  | BacR [ME L^-1^] | 1619 | 181 | 15-8430 | 2955 |  | BacR [ME L^-1^] | 149026 | 48850 | 2220-820000 | 221658 |
|  | BacH [ME L^-1^] | 45.9 | 0.0 | 0-328 | 109.5 |  | BacH [ME L^-1^] | 86.7 | 0.0 | 0-383 | 128.3 |

**Supplementary Table 3.** Summary table depicting ratios for different taxonomic groups or physical and chemical standard parameters between high discharge event (EV) and baseflow (BF) samples. Ratios between mean values of event (EV) and baseflow (BF) samples depict overall changes, while ratios between the maximum abundances/concentration during the event (EV) and mean abundance/concentration during baseflow (BF) conditions are used as a measure to assess the indicator capacity of the respective bacterial group or parameter for high-discharge or surface-influence. Absolute abundances (*) for bacterial groups refer to estimated cell concentrations per mL as calculated by multiplication of the relative proportion of the respective bacterial group with TCC [cells/mL]. BF … baseflow samples; EV … high-discharge event samples.

| **(Candidate) Phylum** | **Class** | **Relative abundances** | | **Absolute abundances*** | |
| --- | --- | --- | --- | --- | --- |
|  |  | **meanEV/ meanBF** | **maxEV/ meanBF** | **meanEV/ meanBF** | **MaxEV/ meanBF** |
| **A** | **-** | **[%]** | **[%]** |  |  |
| Bacteroidetes | - | 1.5 | 3.3 | - | - |
| SM2F11 | - | 1.6 | 2.0 | - | - |
| Parcubacteria | - | 1.3 | 1.6 | - | - |
| Gracilibacteria | - | 1.0 | 1.4 | - | - |
| Actinobacteria | - | 0.6 | 1.0 | - | - |
| Proteobacteria | - | 0.8 | 1.0 | - | - |
| Nitrospirae | - | 0.3 | 0.8 | - | - |
|  |  |  |  |  |  |
| **B** |  | **[%]** | **[%]** | **[× 10^3^ mL^-1^]** | **[× 10^3^ mL^-1^]** |
| Bacteroidetes | Flavobacteriia | 2.2 | 5.5 | 5.5 | 19.2 |
| Proteobacteria | Gammaproteobacteria | 1.1 | 1.5 | 4.2 | 11.9 |
| SM2F11 | uncultured bacterium | 1.6 | 2.0 | 4.6 | 11.3 |
| Gracilibacteria | uncultured sludge bacterium | 1.4 | 1.9 | 4.2 | 9.8 |
| Parcubacteria | uncultured bacterium | 1.3 | 1.6 | 3.7 | 7.9 |
| Proteobacteria | Alphaproteobacteria | 0.8 | 1.1 | 3.1 | 7.7 |
| Parcubacteria | bacterium SH4-10 | 1.4 | 1.8 | 4.1 | 7.6 |
| Gracilibacteria | uncultured bacterium | 1.1 | 1.7 | 3.0 | 6.0 |
| Proteobacteria | Deltaproteobacteria | 1.0 | 1.3 | 2.6 | 5.5 |
| Bacteroidetes | Sphingobacteriia | 1.0 | 1.9 | 2.4 | 4.9 |
| Gracilibacteria | Other | 0.9 | 1.2 | 2.4 | 4.7 |
| Acidobacteria | Acidobacteria | 0.8 | 1.9 | 2.0 | 4.1 |
| Gracilibacteria | uncultured bacterium GKS2-174 | 0.7 | 1.0 | 2.0 | 3.5 |
| Other | Other | 0.7 | 0.9 | 1.8 | 3.5 |
| Proteobacteria | Betaproteobacteria | 0.6 | 0.9 | 1.6 | 3.2 |
| Actinobacteria | Actinobacteria | 0.6 | 0.9 | 1.3 | 2.2 |
| Nitrospirae | Nitrospira | 0.3 | 0.8 | 0.8 | 1.6 |
|  |  |  |  |  |  |
| **C** | **Measurement unit** |  |  |  |  |
| SAC_254_ [abs m^-1^] | | - | - | 6.4 | 9.9 |
| Turbidity [FNU] | | - | - | 9.1 | 15.8 |
| *E. coli* [CFU L^-1^] | | - | - | 28.3 | 100.3 |
| Enterococci [CFU L^-1^] | | - | - | 48.5 | 207.2 |
| Total Coliforms [CFU L^-1^] | | - | - | 28.8 | 111.0 |
| HPC22 [CFU mL^-1^] | | - | - | 32.2 | 143.8 |
| HPC37 [CFU mL^-1^] | | - | - | 50.9 | 227.5 |
| BacR [ME L^-1^] | | - | - | 92.0 | 506.3 |
| BacH [ME L^-1^] | | - | - | 1.9 | 8.3 |

**Supplementary Table 4.** Summary of results from an IMNGS-analysis for the characterization of the two most abundant *Flavobacteriia*-OTUs (OTU_6 & OTU_7) – both affiliated to the genus *Flavobacterium* – according to their occurrence in a variety of publicly available NGS-libraries deposited in public repositories (short read archives (SRA) <https://www.imngs.org/>; Lagkouvardos et al., 2016). (A) shows occurrences of the two OTUs of interest in samples from different selected habitats (‘Environmental sources’) for which hits were obtained (without any threshold of a minimum required occurrence in each sample). (B) shows the detailed analysis results including relative abundances of the closest related sequences in different ‘Environmental source’ samples. Calculations are based on results obtained using a 97% similarity threshold to blast the query sequences (representative sequences of OTU_6 & OTU_7) against publicly available amplicon libraries of the V1-V2 region of the bacterial 16S rRNA gene).

| **A**: Occurrence of closely related sequences of two Flavobacterium-affiliated OTUs (OTU_6 & OTU_7) in samples classified according to their environmental source in the IMNGS-database. | | | | |
| --- | --- | --- | --- | --- |
| **‘Environmental source’ according to IMNGS** | **OTU-name** | **total # of included samples for respective environmental source** | **total # of samples in which respective OTU could be detected** | **Proportion of samples with occurrence of respective OTU* [%]** |
| **freshwater metagenome** | OTU_6 | **5820** | **88** | **1.51** |
|  | OTU_7 | **5820** | **15** | **0.26** |
| lake water metagenome | OTU_6 | 376 | 5 | 1.33 |
| aquatic metagenome | OTU_6 | 3744 | 4 | 0.11 |
| riverine metagenome | OTU_7 | 274 | 2 | 0.73 |
| freshwater sediment metagenome | OTU_6 | 1106 | 1 | 0.09 |
|  | OTU_7 | 1106 | 1 | 0.09 |
| **soil metagenome** | OTU_6 | **29894** | **1** | **0.00** |
|  | OTU_7 | **29894** | **1** | **0.00** |
|  | | | | |
| **B**: Detailed information on the (quantitative) occurrence of OTU_6 (Uniq6) and OTU_7 (Uniq7) | | | | |
| **Description** | | | **Absolute numbers** | **%** |
| **OTU_6 detailed analysis** | | |  |  |
| OTU_6 matches (97%) in all included libraries | | | 781 |  |
|  | | |  |  |
| OTU_6 matches (97%) in 'freshwater metagenome' libraries | | | 384 | 49.2 |
| Max. rel. abundance of OTU_6 in a 'freshwater metagenome' library | | |  | 2.4 |
| Mean. rel. abundance of OTU_6 in a 'freshwater metagenome' library | | |  | 0.2 |
|  | | |  |  |
| OTU_6 matches (97%) in 'soil metagenome' libraries | | | 13 | 1.7 |
| Max. rel. abundance of OTU_6 in a 'soil metagenome' library | | |  | 0.0 |
| Mean. rel. abundance of OTU_6 in a 'soil metagenome' library | | |  | - |
| **OTU_7 detailed analysis** | | |  |  |
| OTU_7 matches (97%) in all libraries(1) | | | 342 |  |
|  | | |  |  |
| OTU_7 matches (97%) in 'freshwater metagenome' libraries | | | 42 | 12.3 |
| Max. rel. abundance of OTU_7 in a 'freshwater metagenome' library | | |  | 0.0 |
| Mean. rel. abundance of OTU_7 in a 'freshwater metagenome' library | | |  | 0.0 |
|  | | |  |  |
| OTU_7 matches (97%) in 'soil metagenome' libraries | | | 1 | 0.3 |
| Max. rel. abundance of OTU_7 in a 'soil metagenome' library | | |  | 0.0 |
| Mean. rel. abundance of OTU_7 in a 'soil metagenome' library | | |  | - |
|  |  |  |  |  |
| **Background information** | | |  |  |
| Description | | | Numbers (Proportions) | |
| Total # of sequences included in analysis | | | 10,767,412,375 | |
| Total # of NGS-libraries (samples) included in analysis | | | 266,675 | |
| Number of 'freshwater metagenome' libraries included | | | 5820 (2.2% of all libraries) | |
| Number of 'soil metagenome' libraries included | | | 29894 (11.2% of all libraries) | |

**Supplementary Table 5.** Barcode and primer sequences for all samples included in this study.

| **Sample_ID** | **Barcode Sequence*** (5‘-3‘) | **Linker-Primer338-Sequence** (5‘-3‘) | **Roche Adapter „Primer A“** (5‘-3‘) | **Primer8F-Sequence** (5‘-3‘) |
| --- | --- | --- | --- | --- |
| BF01 | AACGCACGCTAG | CATGCTGCCTCCCGTAGGAGT | GCCTCCCTCGCGCCATCAG | AGAGTTTGATCCTGGCTCAG |
| BF02 | ACTCACGGTATG | CATGCTGCCTCCCGTAGGAGT | GCCTCCCTCGCGCCATCAG | AGAGTTTGATCCTGGCTCAG |
| BF03 | AGAGCAAGAGCA | CATGCTGCCTCCCGTAGGAGT | GCCTCCCTCGCGCCATCAG | AGAGTTTGATCCTGGCTCAG |
| BF03.R2 | ACCAGCGACTAG | CATGCTGCCTCCCGTAGGAGT | GCCTCCCTCGCGCCATCAG | AGAGTTTGATCCTGGCTCAG |
| BF04 | ACATGATCGTTC | CATGCTGCCTCCCGTAGGAGT | GCCTCCCTCGCGCCATCAG | AGAGTTTGATCCTGGCTCAG |
|  | ACATGTCACGTG | CATGCTGCCTCCCGTAGGAGT | GCCTCCCTCGCGCCATCAG | AGAGTTTGATCCTGGCTCAG |
|  | ACATTCAGCGCA | CATGCTGCCTCCCGTAGGAGT | GCCTCCCTCGCGCCATCAG | AGAGTTTGATCCTGGCTCAG |
|  | ACCACATACATC | CATGCTGCCTCCCGTAGGAGT | GCCTCCCTCGCGCCATCAG | AGAGTTTGATCCTGGCTCAG |
|  | ACCAGACGATGC | CATGCTGCCTCCCGTAGGAGT | GCCTCCCTCGCGCCATCAG | AGAGTTTGATCCTGGCTCAG |
|  | ACTCTTCTAGAG | CATGCTGCCTCCCGTAGGAGT | GCCTCCCTCGCGCCATCAG | AGAGTTTGATCCTGGCTCAG |
|  | ACTGACAGCCAT | CATGCTGCCTCCCGTAGGAGT | GCCTCCCTCGCGCCATCAG | AGAGTTTGATCCTGGCTCAG |
|  | ACTGATCCTAGT | CATGCTGCCTCCCGTAGGAGT | GCCTCCCTCGCGCCATCAG | AGAGTTTGATCCTGGCTCAG |
|  | ACTGTACGCGTA | CATGCTGCCTCCCGTAGGAGT | GCCTCCCTCGCGCCATCAG | AGAGTTTGATCCTGGCTCAG |
| BF05 | ACGCGATACTGG | CATGCTGCCTCCCGTAGGAGT | GCCTCCCTCGCGCCATCAG | AGAGTTTGATCCTGGCTCAG |
| BF06 | ACCTCGATCAGA | CATGCTGCCTCCCGTAGGAGT | GCCTCCCTCGCGCCATCAG | AGAGTTTGATCCTGGCTCAG |
| BF06.R2 | AGCACGAGCCTA | CATGCTGCCTCCCGTAGGAGT | GCCTCCCTCGCGCCATCAG | AGAGTTTGATCCTGGCTCAG |
| BF07 | ACTCAGATACTC | CATGCTGCCTCCCGTAGGAGT | GCCTCCCTCGCGCCATCAG | AGAGTTTGATCCTGGCTCAG |
| BF08 | AAGCTGCAGTCG | CATGCTGCCTCCCGTAGGAGT | GCCTCCCTCGCGCCATCAG | AGAGTTTGATCCTGGCTCAG |
| EV01 | AATCAGTCTCGT | CATGCTGCCTCCCGTAGGAGT | GCCTCCCTCGCGCCATCAG | AGAGTTTGATCCTGGCTCAG |
| EV02 | AATCGTGACTCG | CATGCTGCCTCCCGTAGGAGT | GCCTCCCTCGCGCCATCAG | AGAGTTTGATCCTGGCTCAG |
| EV03 | ACACACTATGGC | CATGCTGCCTCCCGTAGGAGT | GCCTCCCTCGCGCCATCAG | AGAGTTTGATCCTGGCTCAG |
| EV04 | ACACATGTCTAC | CATGCTGCCTCCCGTAGGAGT | GCCTCCCTCGCGCCATCAG | AGAGTTTGATCCTGGCTCAG |
| EV05 | ACACGAGCCACA | CATGCTGCCTCCCGTAGGAGT | GCCTCCCTCGCGCCATCAG | AGAGTTTGATCCTGGCTCAG |
| EV06 | ACGTACTCAGTG | CATGCTGCCTCCCGTAGGAGT | GCCTCCCTCGCGCCATCAG | AGAGTTTGATCCTGGCTCAG |
| EV06.R2 | AGCAGTCGCGAT | CATGCTGCCTCCCGTAGGAGT | GCCTCCCTCGCGCCATCAG | AGAGTTTGATCCTGGCTCAG |
| EV07 | ACGTCTGTAGCA | CATGCTGCCTCCCGTAGGAGT | GCCTCCCTCGCGCCATCAG | AGAGTTTGATCCTGGCTCAG |
| EV08 | ACGTGAGAGAAT | CATGCTGCCTCCCGTAGGAGT | GCCTCCCTCGCGCCATCAG | AGAGTTTGATCCTGGCTCAG |
| EV09 | ACGTGCCGTAGA | CATGCTGCCTCCCGTAGGAGT | GCCTCCCTCGCGCCATCAG | AGAGTTTGATCCTGGCTCAG |
| EV10 | ACACGGTGTCTA | CATGCTGCCTCCCGTAGGAGT | GCCTCCCTCGCGCCATCAG | AGAGTTTGATCCTGGCTCAG |
| EV11 | ACACTAGATCCG | CATGCTGCCTCCCGTAGGAGT | GCCTCCCTCGCGCCATCAG | AGAGTTTGATCCTGGCTCAG |
| EV12 | ACACTGTTCATG | CATGCTGCCTCCCGTAGGAGT | GCCTCCCTCGCGCCATCAG | AGAGTTTGATCCTGGCTCAG |
| EV12.R2 | ACGTTAGCACAC | CATGCTGCCTCCCGTAGGAGT | GCCTCCCTCGCGCCATCAG | AGAGTTTGATCCTGGCTCAG |
| EV13 | ACAGACCACTCA | CATGCTGCCTCCCGTAGGAGT | GCCTCCCTCGCGCCATCAG | AGAGTTTGATCCTGGCTCAG |
| EV14 | ACAGAGTCGGCT | CATGCTGCCTCCCGTAGGAGT | GCCTCCCTCGCGCCATCAG | AGAGTTTGATCCTGGCTCAG |
| EV14.R2 | ACTACAGCCTAT | CATGCTGCCTCCCGTAGGAGT | GCCTCCCTCGCGCCATCAG | AGAGTTTGATCCTGGCTCAG |
| EV15 | ACAGCAGTGGTC | CATGCTGCCTCCCGTAGGAGT | GCCTCCCTCGCGCCATCAG | AGAGTTTGATCCTGGCTCAG |
| EV16 | ACAGCTAGCTTG | CATGCTGCCTCCCGTAGGAGT | GCCTCCCTCGCGCCATCAG | AGAGTTTGATCCTGGCTCAG |
| BF09 | ACAGTGCTTCAT | CATGCTGCCTCCCGTAGGAGT | GCCTCCCTCGCGCCATCAG | AGAGTTTGATCCTGGCTCAG |
| BF10 | AGCGCTGATGTG | CATGCTGCCTCCCGTAGGAGT | GCCTCCCTCGCGCCATCAG | AGAGTTTGATCCTGGCTCAG |
|  | AGCGTAGGTCGT | CATGCTGCCTCCCGTAGGAGT | GCCTCCCTCGCGCCATCAG | AGAGTTTGATCCTGGCTCAG |
| BF11 | AACTGTGCGTAC | CATGCTGCCTCCCGTAGGAGT | GCCTCCCTCGCGCCATCAG | AGAGTTTGATCCTGGCTCAG |
| BF12 | ACGCTATCTGGA | CATGCTGCCTCCCGTAGGAGT | GCCTCCCTCGCGCCATCAG | AGAGTTTGATCCTGGCTCAG |
| BF12.R2 | AGCAGCACTTGT | CATGCTGCCTCCCGTAGGAGT | GCCTCCCTCGCGCCATCAG | AGAGTTTGATCCTGGCTCAG |
| BF13 | ACTATTGTCACG | CATGCTGCCTCCCGTAGGAGT | GCCTCCCTCGCGCCATCAG | AGAGTTTGATCCTGGCTCAG |
| BF14 | ACGCTCATGGAT | CATGCTGCCTCCCGTAGGAGT | GCCTCCCTCGCGCCATCAG | AGAGTTTGATCCTGGCTCAG |
| BF15 | ACGGATCGTCAG | CATGCTGCCTCCCGTAGGAGT | GCCTCCCTCGCGCCATCAG | AGAGTTTGATCCTGGCTCAG |
| BF16 | AGATGTTCTGCT | CATGCTGCCTCCCGTAGGAGT | GCCTCCCTCGCGCCATCAG | AGAGTTTGATCCTGGCTCAG |

**REFERENCES**

Berry, D., Ben Mahfoudh, K., Wagner, M., and Loy, A. (2011). Barcoded Primers Used in Multiplex Amplicon Pyrosequencing Bias Amplification. *Applied and Environmental Microbiology* 77(21)**,** 7846-7849. doi: 10.1128/aem.05220-11.

Fierer, N., Hamady, M., Lauber, C.L., and Knight, R. (2008). The influence of sex, handedness, and washing on the diversity of hand surface bacteria. *Proceedings of the National Academy of Sciences* 105(46)**,** 17994-17999. doi: 10.1073/pnas.0807920105.

Frank, D.N., St. Amand, A.L., Feldman, R.A., Boedeker, E.C., Harpaz, N., and Pace, N.R. (2007). Molecular-phylogenetic characterization of microbial community imbalances in human inflammatory bowel diseases. *Proceedings of the National Academy of Sciences* 104(34)**,** 13780-13785. doi: 10.1073/pnas.0706625104.

Yilmaz, P., Parfrey, L.W., Yarza, P., Gerken, J., Pruesse, E., Quast, C., et al. (2014). The SILVA and “All-species Living Tree Project (LTP)” taxonomic frameworks. *Nucleic Acids Research* 42(D1)**,** D643-D648. doi: 10.1093/nar/gkt1209.
